# Kun-Ling Wan Formula Ameliorates Postmenopausal Osteoporosis and Adipose Accumulation by Suppressing mTOR Signaling in Mesenchymal Stem Cells

**DOI:** 10.3390/ph19050719

**Published:** 2026-04-30

**Authors:** Xiaoqing Lu, Tingting Xie, He Lan, Yaqi Fan, Jie Yang, Qianzan Liao, Yuxin Jin, Yaoxuan Zhu, Jingxin Zhang, Dexin Li, Chunshui Pan, Quan Li, Kai Sun, Xinmei Huo, Ting Yuwen, Jing-Yan Han, Yin Li

**Affiliations:** 1 Department of Integration of Chinese and Western Medicine, School of Basic Medical Sciences, Peking University, 38 Xueyuan Road, Haidian District, Beijing 100191, China; xiaoqinglu@bjmu.edu.cn (X.L.); xiett@bjmu.edu.cn (T.X.); shellyfann@163.com (Y.F.); 1610305115@pku.edu.cn (Q.L.); jinyx@bjmu.edu.cn (Y.J.); zhuyaoxuan@bjmu.edu.cn (Y.Z.); may_zhang@stu.pku.edu.cn (J.Z.); lidexin17@mails.ucas.ac.cn (D.L.); audeline@sina.com (X.H.); 2 Tasly Microcirculation Research Center, Peking University Health Science Center, Beijing 100191, China; 2311210292@stu.pku.edu.cn (J.Y.); chunshuipan2002@163.com (C.P.); liquan213@126.com (Q.L.); kais211@163.com (K.S.); 3 Department of Physiology and Pathophysiology, School of Basic Medical Sciences, Peking University, Beijing 100191, China; bylanhe08@126.com; 4 Department of Clinical Laboratory, Xuanwu Hospital, Capital Medical University, Beijing 100053, China; 5 National Institute on Drug Dependence and Beijing Key Laboratory of Drug Dependence Research, Peking University, Beijing 100191, China; yuwenting@bjmu.edu.cn

**Keywords:** postmenopausal osteoporosis, fat accumulation, mesenchymal stem cells, osteogenesis, adipogenesis, mTOR pathway, Kun-Ling Wan Formula

## Abstract

**Background**: Postmenopausal osteoporosis is a common metabolic bone disorder characterized by decreased bone mass and microstructural deterioration, often accompanied by increased bone marrow adiposity and systemic fat accumulation. Kun-Ling Wan Formula (KLW) is a compound Chinese medicine clinically used for gynecological disorders, though its effects on postmenopausal osteoporosis and associated fat accumulation remain unclear. Distinct from previous herbal formulation studies that primarily focused on bone outcomes, our study uniquely integrates bone protection, marrow adiposity reduction, systemic metabolic improvement, and multi-omics mechanistic dissection in a high-fat diet-fed ovariectomized mouse model. **Methods**: KLW chemical composition was analyzed by UPLC-Q-TOF/MS. Ovariectomized (OVX) C57BL/6J mice fed high-fat or normal diet were treated with KLW at clinically equivalent or double doses, with estrogen and active compounds as controls. Bone microstructure was assessed by micro-CT, bone marrow fat by MRI-PDFF, and metabolism by OGTT, ITT, and metabolic cages. Network pharmacology, proteomics, molecular docking, and dynamics simulations identified core targets. C3H10T1/2 cells were used to assess osteogenic/adipogenic differentiation and mTOR pathway activation. **Results**: Twelve compounds were identified in KLW. In OVX mice, KLW significantly improved bone mineral density and trabecular microstructure, reduced adiposity and bone marrow fat, and enhanced glucose tolerance and insulin sensitivity. In vitro, KLW promoted osteogenesis and suppressed adipogenesis in C3H10T1/2 cells. Integrative analyses identified mTOR as a central target, with chrysophanol, pyrogallol, and apigenin showing high-affinity binding. KLW inhibited mTOR/S6K phosphorylation during differentiation, an effect reversible by leucine. **Conclusions**: KLW ameliorates osteoporosis and reduces fat accumulation in OVX mice by shifting mesenchymal stem cell differentiation toward osteogenesis via mTOR pathway modulation.

## 1. Introduction

Postmenopausal osteoporosis, characterized by reduced bone mineral density (BMD) and deteriorated bone structure, significantly impacts a person’s quality of life and increases their risk of fractures [1,2]. In China, it is estimated that osteoporosis-related fractures will triple by 2050 and, as such, this represents a significant future economic and healthcare burden [3]. Estrogen deficiency, the primary cause of postmenopausal osteoporosis, disrupts the balance between bone resorption and formation, leading to accelerated bone loss. Moreover, this pathological process is exacerbated by increased fat accumulation, a phenomenon frequently observed in postmenopausal women due to metabolic dysregulation [4].

Despite being a cornerstone treatment for postmenopausal osteoporosis, estrogen replacement therapy (ERT) is associated with significant risks that limit its clinical utility and long-term safety as a treatment for this condition [5]. Numerous large-scale studies have established a strong association between exogenous estrogen administration and increased incidences of adverse effects, including breast cancer [6], endometrial hyperplasia [7], thromboembolic events [8], and cardiovascular complications [5]. Arguably, such safety concerns indicate that ERT may be a suboptimal choice for postmenopausal women, particularly those with a history of hormone-dependent cancers or predisposing risk factors. As such, the identification and use of alternative therapeutic strategies that offer beneficial effects of estrogen therapy without such off-target effects remains a significant unmet need [9].

The cellular basis for postmenopausal osteoporosis remains understudied; however, the actions of mesenchymal stem cells (MSCs) and their capacity to differentiate into osteoblasts or adipocytes are known to be critical to maintaining bone homeostasis in this context. In postmenopausal women, MSCs exhibit impaired osteogenic differentiation and enhanced adipogenic differentiation, contributing to decreased bone formation and increased marrow fat [10]. Of the cellular signalling mechanisms involved, the mammalian target of rapamycin (mTOR) signaling pathway is crucial as a regulator of MSC differentiation [11]. In the context of nutrient excess or high-energy states, overactivation of mechanistic target of rapamycin complex 1 (mTORC1) promotes adipogenesis while inhibiting osteogenesis, thereby exacerbating bone loss and fat accumulation in this setting. Targeting this pathway offers a potential therapeutic strategy to address such pathological traits simultaneously [11].

Kun-Ling Wan Formula (KLW), a compound traditional Chinese medicine, has been used clinically for treating estrogen-deficiency-related disorders such as infertility and polycystic ovary syndrome. Comprising a formulation derived from 31 herbal components (Table S1), the plant species names have been verified using the World Flora Online database https://www.worldfloraonline.org (accessed on 27 October 2025). KLW is known for its multifaceted therapeutic properties, including exerting hormone regulation and anti-inflammatory effects [12]. However, its therapeutic potential for postmenopausal osteoporosis and fat accumulation remains unexplored. Here, we have investigated the effects of KLW treatment in ovariectomized (OVX) mice, a preclinical model of postmenopausal pathologies. To contextualize our study of the efficacy of treatment with KLW which is a compound formulation, we conducted parallel experiments using two well-characterized natural phytochemicals, namely psoralen (PSO) and asperosaponin VI (ASP), as positive controls. Both PSO, a major bioactive component of Psoralea corylifolia [13], as well as ASP, a principal saponin derived from Dipsacus asperoides [14], have been extensively reported to ameliorate bone loss in ovariectomized rodents by enhancing osteogenic activity and inhibiting osteoclastic bone resorption [14,15,16,17,18,19,20]. Their inclusion in this study design is relevant as they represent gold-standard natural agents in anti-osteoporosis research, such that we assess the bone-protective effects of KLW alongside treatment through these control experiments to benchmark our findings.

## 2. Results

### 2.1. Identification of KLW Components in Circulating Blood

**Figure 1:**
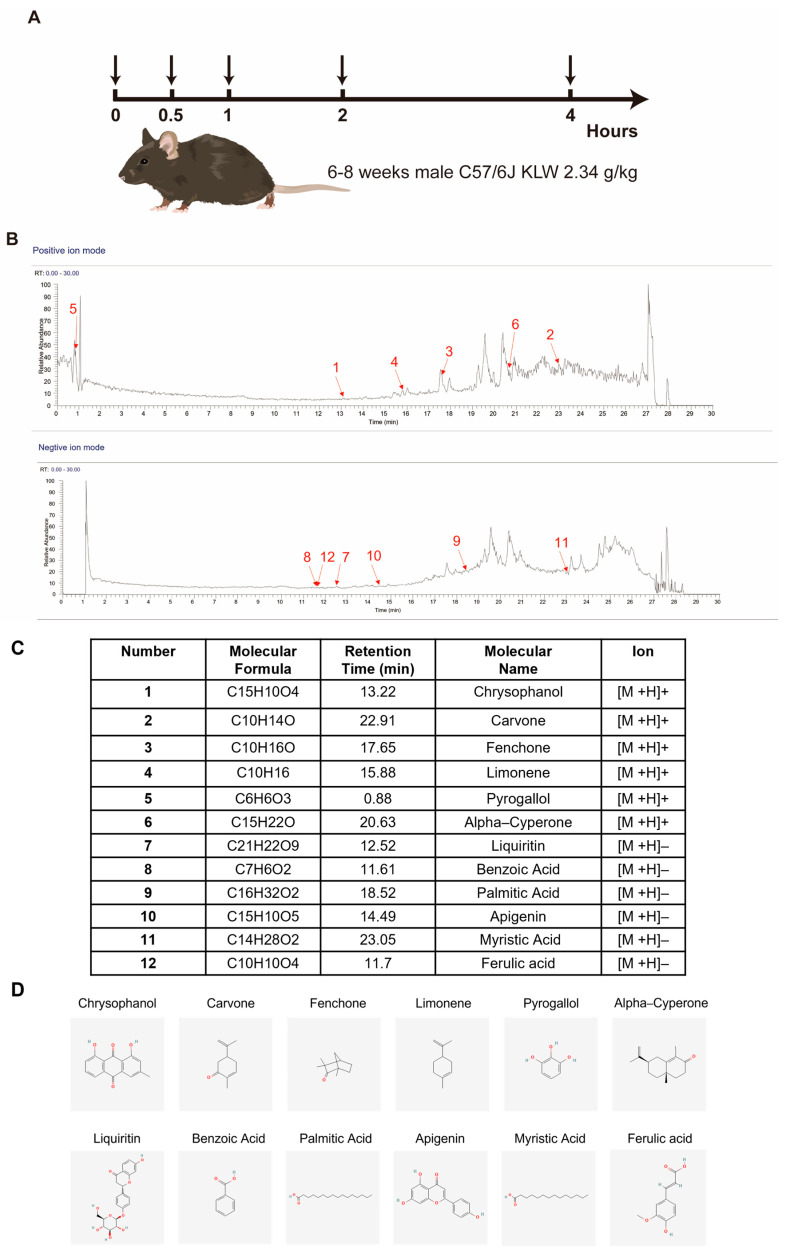
Identification of KLW components in the sera of treated mice. (**A**) Schematic diagram of the technical workflow for detecting KLW components in blood. (**B**,**C**) Base peak chromatogram of prototype components of KLW absorbed into the blood detected by UPLC/Q–TOF–MS in positive-ion and negative ion mode. (**D**) Chemical structures of the components.

To investigate the components and underlying effects of Kun-Ling Wan Formula (KLW) in the bloodstream of treated mice, serum samples were collected from mice treated with KLW (2.34 g/kg) at 0, 0.5, 1, 2, and 4 h after gavage (Figure 1A). All the components of KLW were measured by quadrupole-time-of-flight mass spectrometry (UPLC/Q-TOF-MS) at retention time (0–30 min) in positive-ion and negative-ion mode. We profiled our results based on our understanding of the main ingredients of KLW, summarized in the TCMSP (Traditional Chinese Medicine Systems Pharmacology Database and Analysis Platform) and information from previous reports. In this way, we developed chromatograms and mass spectra linked to these previously characterized components across different timepoints, illustrated using Xcalibur software (version 4.0) (Figure 1B). As shown, our results demonstrated that 12 prototype components of KLW were detected in the blood of treated mice (Figure 1C,D). To ensure the reproducibility of our findings, we assessed the batch-to-batch consistency of KLW. No significant batch-to-batch variation was observed among the 11 lots of KLW tested (Figure S1A).

### 2.2. KLW Improved Bone Health in OVX Mice Under High-Fat Diet

**Figure 2:**
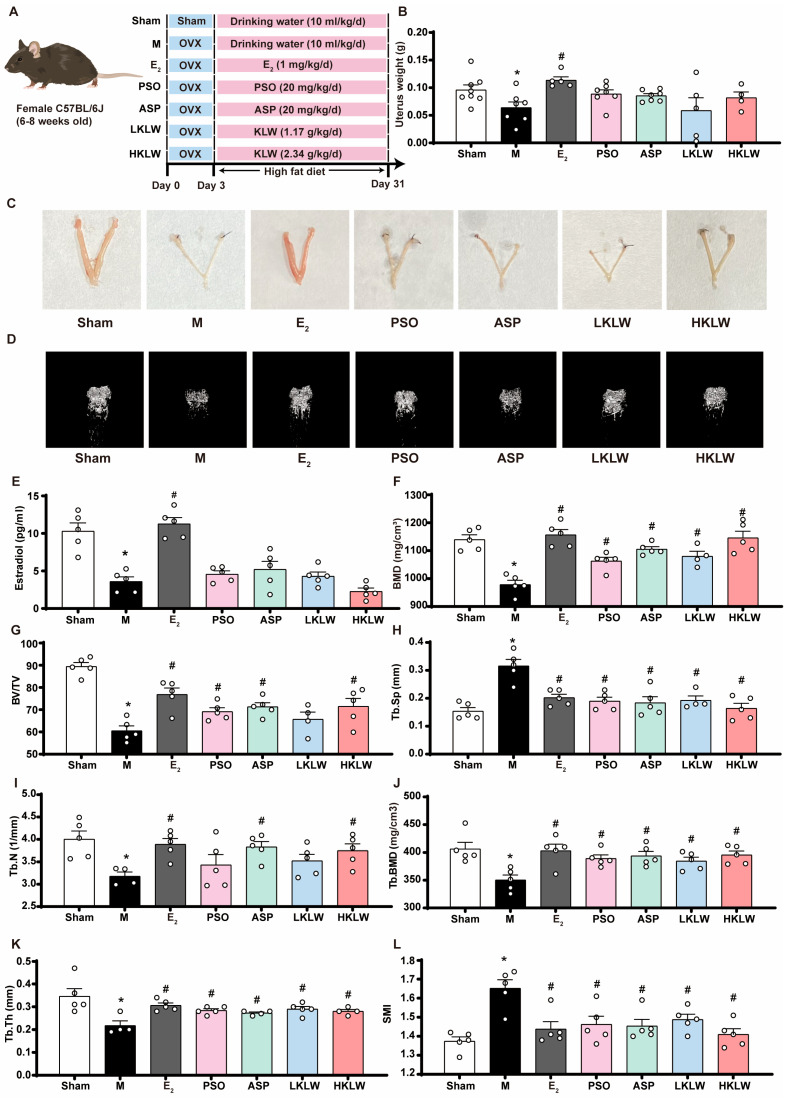
KLW improved bone health in OVX mice. Mice were randomly assigned to undergo either sham surgery or OVX. After a 3–day postoperative recovery period, all animals were fed a HFD throughout the experimental period. Upon initiation of the diet, mice received daily gavage treatments for 28 consecutive days of the following treatments: Sham (drinking water), M (drinking water), E_2_ (estrogen), PSO (psoralen), ASP (asperosaponin VI), LKLW (low dose KLW), and HKLW (high dose KLW), respectively (see Methods for details). (**A**) Schematic diagram of treatment groups and drug regimen. (**B**) Uterine wet weight; (**C**) Representative gross morphological images of uterine tissues. (**D**) Three-dimensional micro-CT reconstructions of the proximal femoral metaphysis of mice. (**E**) Plasma estradiol levels. (**F**–**L**) Quantitative analysis of bone microarchitectural parameters from micro-CT. (**F**) bone mineral density (BMD). (**G**) bone volume fraction (BV/TV). (**H**) trabecular separation (Tb.Sp). (**I**) trabecular number (Tb.N). (**J**) trabecular bone density (Tb.BMD). (**K**) trabecular thickness (Tb.Th) and (**L**) structure model index (SMI) measurements. All data are presented as the mean ± SEM. n = 4–5. Statistical analysis was performed using one-way ANOVA followed by Tukey’s post hoc test: * *p* < 0.05 compared to the Sham group, # *p* < 0.05 compared to the M group.

To evaluate the potential effects of KLW on osteoporosis in ovariectomized mice fed a high-fat diet (HFD), 6–8-week-old female C57BL/6J mice were selected and subjected to either sham surgery or bilateral ovariectomy (OVX). Three days post-surgery, all mice were placed on HFD until the end of the experiment. The ovariectomized mice were randomly assigned into several treatment groups where each received daily oral gavage of either estrogen, clinically relevant dosages of anti-osteoporosis phytochemicals psoralen (PSO) and asperosaponin VI (ASP) serving as positive controls, or KLW at low (LKLW) and high doses (HKLW) that corresponds to the clinical equivalent dose and twice that dose, respectively (Figure 2A). We first evaluated the safety profile of each dose. KLW treatment did not induce detectable hepatic or renal toxicity (Figure S1B–E). To assess the success of the model and to determine whether the administered treatments exerted estrogen-like effects, the uteri of mice were collected from each treatment group and weighed (Figure 2B,C and Figure S1F), and this showed that uterine weights in the model group were significant decreased compared to Sham. Moreover, estrogen treatment markedly attenuated uterine atrophy in OVX mice. In contrast, there was no significant difference in uterine weights for OVX mice treated with PSO, ASP, LKLW or HKLW.

To evaluate the impact of KLW treatment on bone microstructure in ovariectomized mice maintained on a HFD, micro-CT analysis was performed on the femora harvested from each experimental group. Three-dimensional reconstruction studies were performed to visualize and quantify the trabecular architecture in the region proximal to the femoral head (Figure 2D, 3D reconstruction animations are provided in Videos S1–S7). As shown, when compared with Sham treatment, the M group exhibited significant bone loss. In contrast, administration of estrogen, PSO, ASP, LKLW, or HKLW significantly ameliorated osteoporotic conditions relative to the M group. Analysis of plasma estrogen levels revealed that LKLW or HLKW treatment did not result in a significant increase in circulating estrogen levels, suggesting that any effects of either dose of KLW may not be mediated through an estrogen-mimetic mechanism (Figure 2E).

Next, we measured bone mineral density (BMD), a key indicator of bone mass and reflective of the degree of osteoporosis and predicts fracture risk, as follows. MicroCT analysis revealed that OVX mice in the M group exhibited a significant decrease in BMD compared to Sham while, in contrast, BMD scores for mice in E_2_, PSO, ASP, LKLW, and HKLW groups were significantly higher than M group mice (Figure 2F). In addition to BMD measurements, we also analyzed bone volume fraction (BV/TV) which reflects bone mass in both cortical and trabecular bone. As shown, while we detected a significant reduction in BV/TV in mice from the M group, BV/TV values for mice from E_2_, PSO, ASP, and HKLW treatment groups, but not LKLW treatment, were significantly higher when compared to M group (Figure 2G). Additionally, we analyzed trabecular separation (Tb.Sp), number (Tb.N), BMD (Tb.BMD) and thickness (Tb.Th) to assess such microstructure features. Samples from the M group showed significantly decreased Tb.N, Tb.BMD and Tb.Th values as well as increased Tb.Sp, compared to samples from the sham group. Such features are characteristic of osteoporotic deterioration in samples from the M group. Treatment with E_2_, PSO, ASP, and KLW markedly ameliorated these osteoporosis-related parameters (Figure 2H–K).

Finally, we measured the structure model index (SMI) for each sample across all groups, indicative of the plate-to-rod ratio of trabeculae, and found that this metric was significantly increased in the M group, which was suggestive of a shift toward rod-like structures. In contrast, treatment with E_2_, PSO, ASP, LKLW and HKLW all resulted in significant reductions in SMI signals, and this indicated that all treatments could restore plate-like trabecular morphologies in OVX mice (Figure 2L).

### 2.3. KLW Improved Bone Health in OVX Mice Under Normal Chow Diet

We performed parallel experiments to examine the effects of KLW treatment on osteoporosis in ovariectomized mice fed a normal chow diet (NCD). Female C57BL/6J mice, aged 6–8 weeks, were subjected to either sham surgery or bilateral ovariectomy. Three days after surgery, all animals were maintained on a NCD until ovariectomized mice were randomly assigned to treatment with E_2_, PSO, and ASP as positive control experiments, as well as low-dose and high-dose KLW administered daily via oral gavage at similar dosages as delivered to OVX mice on a HFD (Figure S1G). As shown, microCT analysis of murine femora across BV/TV, Tb.Sp, Tb.N, Tb.BMD, Tb.TH and SMI measurements all demonstrated that OVX mice fed a normal diet were significantly impaired in these features compared to sham treatment, while treatment with PSO, ASP, LKLW and HKLW could ameliorate these features that are consistent with osteoporosis in OVX mice (Figure S1H–N). KLW significantly ameliorated osteoporosis in ovariectomized mice under NCD.

### 2.4. KLW Treatment Attenuated Fat Accumulation in High-Fat Diet-Fed OVX Mice

**Figure 3:**
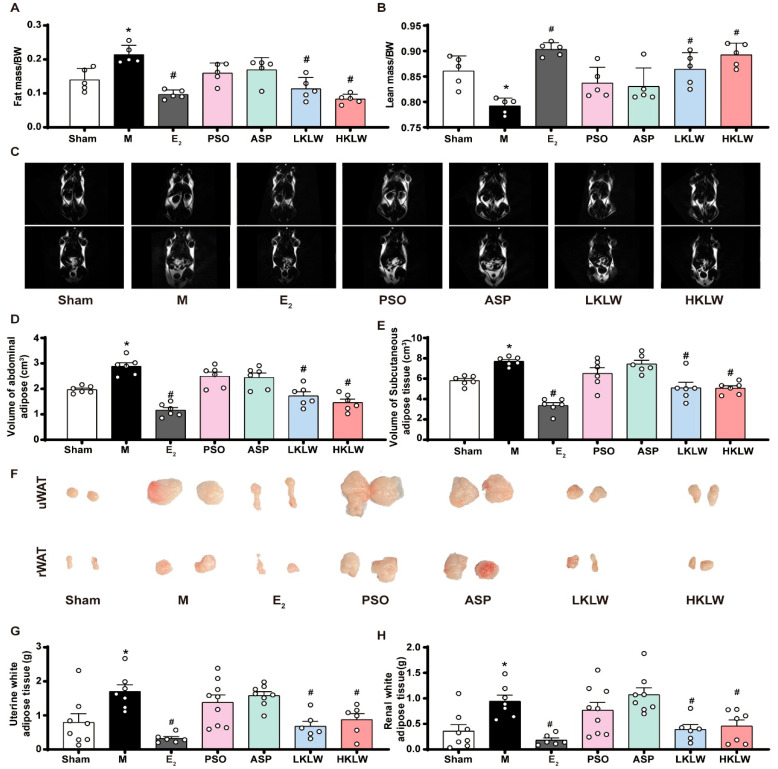
KLW treatment attenuated fat accumulation in high-fat diet-fed OVX mice. (**A**) Statistical graph depicting the ratio of total body fat weight to body weight. (**B**) Statistical graph depicting the ratio of total body lean weight to body weight. (**C**) Representative images of whole-body fat distribution in mice. Adipose tissue was identified as hyperintense (bright) regions on the images. (**D**,**E**) Quantification of abdominal fat and subcutaneous volumes derived from whole-body fat scanning. (**F**) Images of parametrial and perirenal adipose tissue. (**G**) Parauterine adipose tissue weight. (**H**) Perirenal adipose tissue weight. All data are presented as the mean ± SEM. n = 5–9. Statistical analysis was performed using one-way ANOVA followed by Tukey’s post hoc test: * *p* < 0.05 compared to the SHAM group, # *p* < 0.05 compared to the M group.

To systematically evaluate the effects of KLW on body composition, we performed non-invasive body composition analysis to quantify whole-body fat and lean mass in mice. As shown in Figure 3A,B, the M group exhibited a significant increase in fat mass and a decrease in lean mass. In contrast, treatment with E_2_, LKLW, or HKLW ameliorated these alterations, while treatment with PSO and ASP did not have such an effect. No significant differences in overall body weight were observed across all groups throughout the treatment period (Figure S2A,B).

To evaluate the impact of KLW treatment on fat distribution and adiposity more closely in vivo, we performed quantitative analysis of whole-body adipose tissue using non-invasive MRI imaging. MRI analysis revealed that LKLW and HKLW treatment significantly reduced the volume of multiple adipose depots in mice, specifically manifesting as decreased volumes of perirenal, perimetrial, and subcutaneous fat tissues (bright areas in the figure represent adipose tissue) (Figure 3C). Quantitative MRI analysis further demonstrated that KLW significantly reduced the volume of both visceral and subcutaneous adipose tissue (Figure 3D,E), confirming its efficacy in modulating fat distribution. To further validate these MRI findings, we excised and weighed the perimetrial and perirenal adipose tissues from the mice. The results consistently demonstrated that fat depots in M group were significantly increased compared with sham group, while treatment with E_2_, LKLW and HKLW significantly reduced the weight of both fat depots in M group, whereas neither PSO nor ASP produced such effects (Figure 3F–H).

### 2.5. KLW Does Not Alter Body Weight or Body Composition in Ovariectomized Mice Under NCD

Consistent with the findings in OVX mice fed HFD, no significant changes in overall body weight were detected among OVX mice fed NCD during the administration period (Figure S3A,B). However, unlike the HFD mice, there were no significant differences in measurements of body fat percentages (Figure S3C), lean mass (Figure S3D), or in the weights of uterine and renal white adipose tissues (Figure S3E,F) in NCD mice across all treatment groups. Similarly, MRI analyses confirmed that there were no appreciable changes in abdominal and subcutaneous fat volumes between the different treatment groups under NCD conditions (Figure S3G,H). These data indicate that KLW exerts its fat reducing effect specifically in the context of HFD, but not under NCD.

### 2.6. KLW Treatment Reduced Bone Marrow Fat Content in OVX Mice

**Figure 4:**
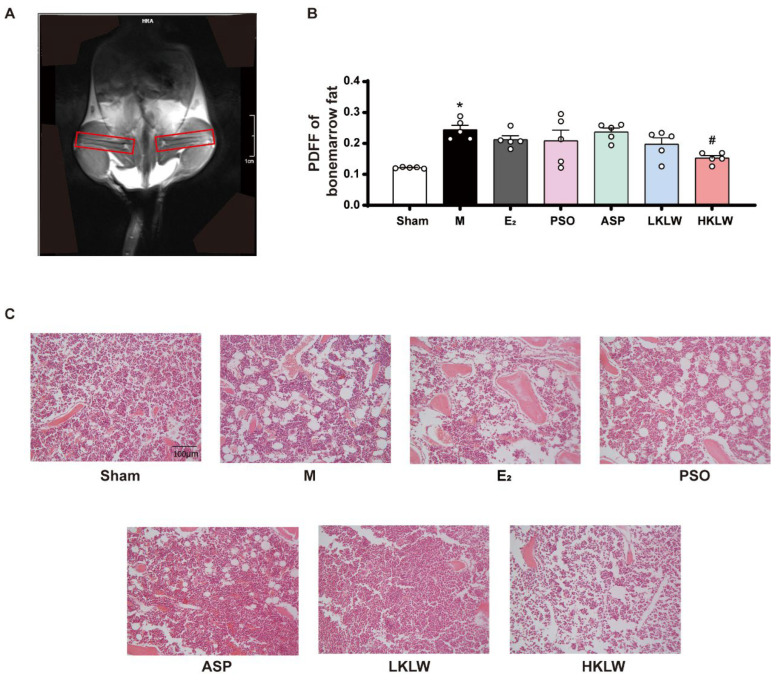
KLW treatment reduced bone marrow fat content in OVX mice. (**A**) Magnetic resonance imaging (MRI) of s representative lower body region of a mouse. The red circles indicate the regions of interest (ROIs) selected for measuring the proton density fat fraction (PDFF) in the bone marrow. (**B**) Statistical results of the proton density fat fraction (PDFF) in the femoral bone marrow of mice. The values represent the average of bilateral measurements. (**C**) Representative H&E staining of femoral bone sections from each experimental group. The scale bar in the images corresponds to 100 μm. All data are presented as the mean ± SEM. n = 5. Statistical analysis was performed using one-way ANOVA followed by Tukey’s post hoc test: * *p* < 0.05 compared to the Sham group, # *p* < 0.05 compared to the M group.

To elucidate the mechanisms by which KLW treatment could ameliorate osteoporosis, the lipid content within the bone marrow of the mouse femur was quantified (Figure 4A). MRI analysis of the lower limbs of mice from each experimental group was conducted. The proton density fat fraction (PDFF) was calculated by selecting the bone marrow regions as indicated in the images, reflecting the lipid content in these areas. The M group exhibited a significant increase in femoral bone marrow lipid content compared to Sham, whereas the HKLW group demonstrated a significant reduction in femoral bone marrow lipid content (Figure 4B). The M group exhibited an increase in both the number and size of bone marrow adipocytes compared to the Sham group. In contrast, the E_2_, PSO, and ASP groups showed no significant changes in adipocyte number or size relative to the M group, while the LKLW and HKLW groups demonstrated a reduction in both the number and size of bone marrow adipocytes compared to the M group (Figure 4C). Likewise, in the NCD mice, both LKLW and HKLW, but not PSO or ASP, attenuated the elevation in bone marrow adiposity induced by OVX compared to sham controls (Figure S4).

### 2.7. KLW Ameliorates HFD-Induced Lipid Accumulation by Restoring Metabolic Homeostasis

**Figure 5:**
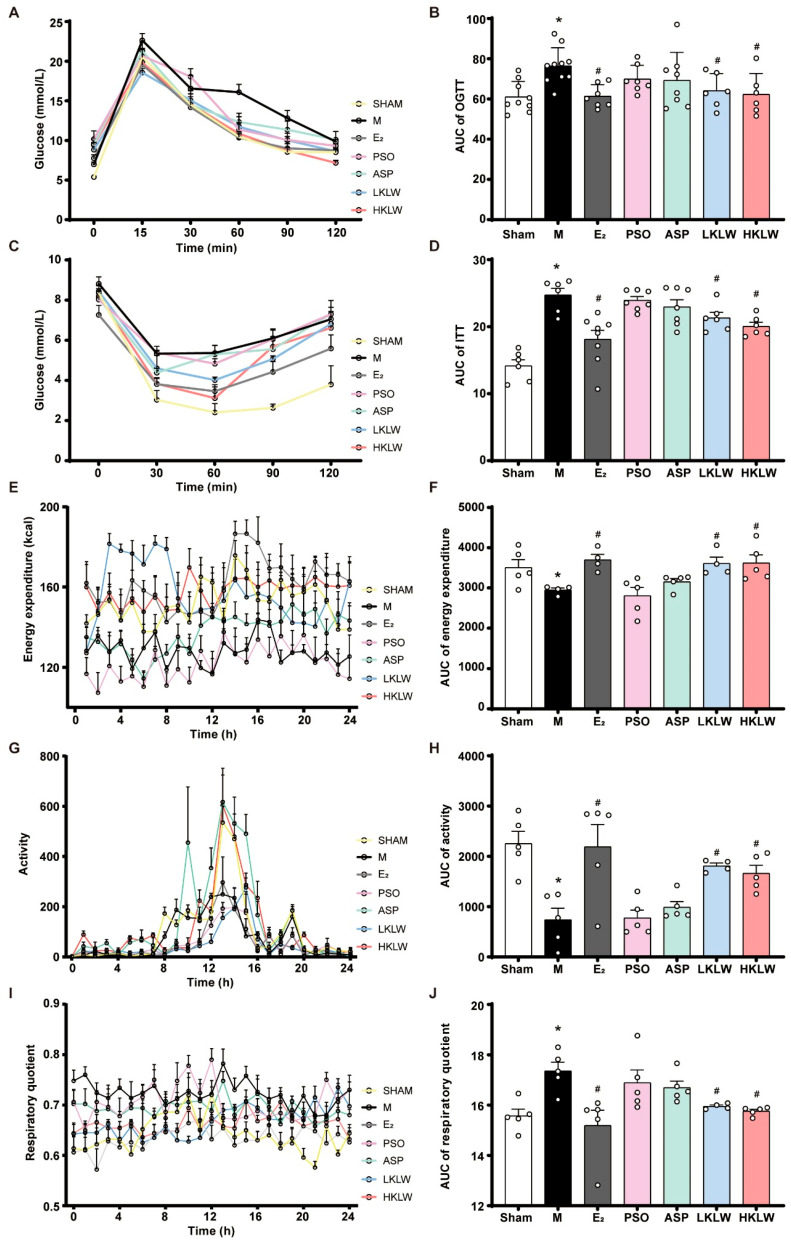
KLW treatment suppresses lipid accumulation induced by OVX and a high-fat diet by modulating metabolic homeostasis. (**A**,**B**) Oral Glucose Tolerance Test (OGTT) and AUC. (**C**,**D**) Insulin Tolerance Test, (ITT) and AUC. (**E**,**F**) Energy expenditure curve and AUC. (**G**,**H**) Activity curve and AUC. (**I**,**J**) Respiratory Exchange Ratio (RER) and AUC. All data are presented as the mean ± SEM. n = 4–10. Statistical analysis was performed using one-way ANOVA followed by Tukey’s post hoc test: * *p* < 0.05 compared to the Sham group, # *p* < 0.05 compared to the M group.

To evaluate the impact of KLW on metabolic regulation in mice, we performed oral glucose tolerance tests (OGTT) and insulin tolerance tests (ITT). As shown in Figure 5A–D, the M group exhibited significantly impaired glucose tolerance and insulin sensitivity compared to the Sham group. Quantitative analysis of the area under the curve (AUC) revealed that the OGTT-AUC was significantly increased in the M group compared to the Sham group. Treatment with LKLW or HKLW significantly reduced the OGTT-AUC compared to the M group. Similarly, the ITT-AUC was significantly elevated in the M group relative to the Sham group, and this elevation was significantly attenuated by treatment with LKLW or HKLW. To further investigate the effects of KLW on systemic energy metabolism, we conducted comprehensive metabolic cage analysis to monitor real-time energy expenditure, physical activity, and respiratory exchange ratio in vivo. As shown treatment with either LKLW or HKLW significantly enhanced energy expenditure and increased physical activity in mice, accompanied by a marked reduction in the respiratory exchange ratio (RER). These results indicated a shift toward increased lipid utilization and improved metabolic efficiency in response to KLW treatment at either dose (Figure 5E–J). Collectively, these results demonstrate that treatment with KLW ameliorates HFD-induced metabolic dysregulation through enhancing catabolic efficiency and substrate utilization.

### 2.8. Effects of KLW on Metabolic Homeostasis in NCD Mice

In NCD mice, although OVX did not exacerbate glucose intolerance, HKLW treatment still significantly improved glucose tolerance (Figure S5A,B). HKLW treatment also counteracted the OVX-induced impairment of insulin sensitivity in NCD-fed mice (Figure S5C,D). However, in NCD mice, there were no significant changes in energy expenditure, physical activity, or respiratory exchange ratio (Figure S5E–J).

### 2.9. KLW-Containing Serum Promotes Osteogenic Differentiation and Suppresses Adipogenic Differentiation in Mesenchymal Stem Cells

**Figure 6:**
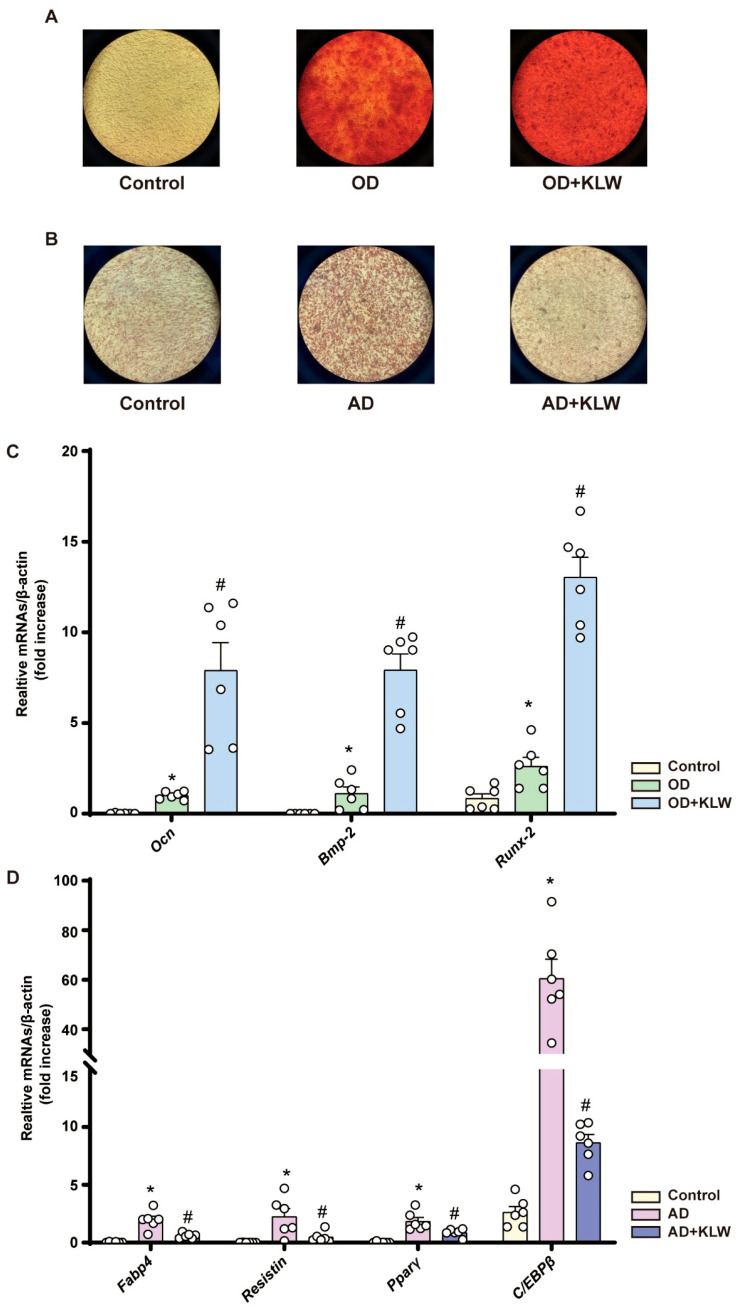
KLW treatment promotes osteogenic differentiation and suppresses adipogenic differentiation in mesenchymal stem cells. (**A**) Alizarin Red S staining. (**B**) Oil Red O staining. (**C**,**D**) Detection of mRNA expression levels by quantitative Real-Time PCR. Control: C3H10T1/2 cells cultured in standard DMEM medium; Differentiation: C3H10T1/2 cells induced with osteogenic/adipogenic differentiation medium; Differentiation + KLW: C3H10T1/2 cells induced with osteogenic/adipogenic differentiation medium supplemented with 10% KLW-containing serum. All data are presented as the mean ± SEM. n = 6. Statistical analysis was performed using one-way ANOVA followed by Tukey’s post hoc test: * *p* < 0.05 compared to the Control group, # *p* < 0.05 compared to the OD/AD group.

The mouse embryonic mesenchymal stem cell line C3H10T1/2 was employed as an experimental model to investigate both osteogenic and adipogenic differentiation processes. Following a 3-day pre-culture to 70–80% confluence, osteogenic differentiation was induced in all groups except the control by switching to a differentiation medium; the group receiving this medium supplemented with KLW was designated the “differentiation + KLW” group. After 7 days of culture in the indicated media, osteogenic differentiation was evaluated by Alizarin Red S staining for calcium nodules, which appear orange-red upon positive staining. Alizarin Red S staining demonstrated a marked increase in calcium nodule formation in the differentiation group compared to the negative control, an effect that was significantly potentiated by the addition of KLW, indicating its promotive role in the osteogenic differentiation of MSCs (Figure 6A).

Following adipogenic induction in overconfluent cultures, Oil Red O staining after 7 days revealed substantial lipid droplet formation, which was markedly attenuated by KLW supplementation. This indicates that KLW-containing serum impairs adipogenic differentiation in MSCs (Figure 6B).

RT-PCR analysis of marker genes after 3 days of differentiation revealed that KLW not only potentiated the upregulation of osteogenic genes (*Ocn*, *Runx2*, *Bmp-2*) but also suppressed the differentiation-induced increase in adipogenic genes (*Fabp4*, *Resistin*, *Pparγ*, *C/Ebpβ*) (Figure 6C,D). These findings indicate that KLW coordinately promotes osteogenic and inhibits adipogenic differentiation pathways.

### 2.10. mTOR Signaling Mediates the Therapeutic Effects of KLW in Mesenchymal Stem Cells

**Figure 7:**
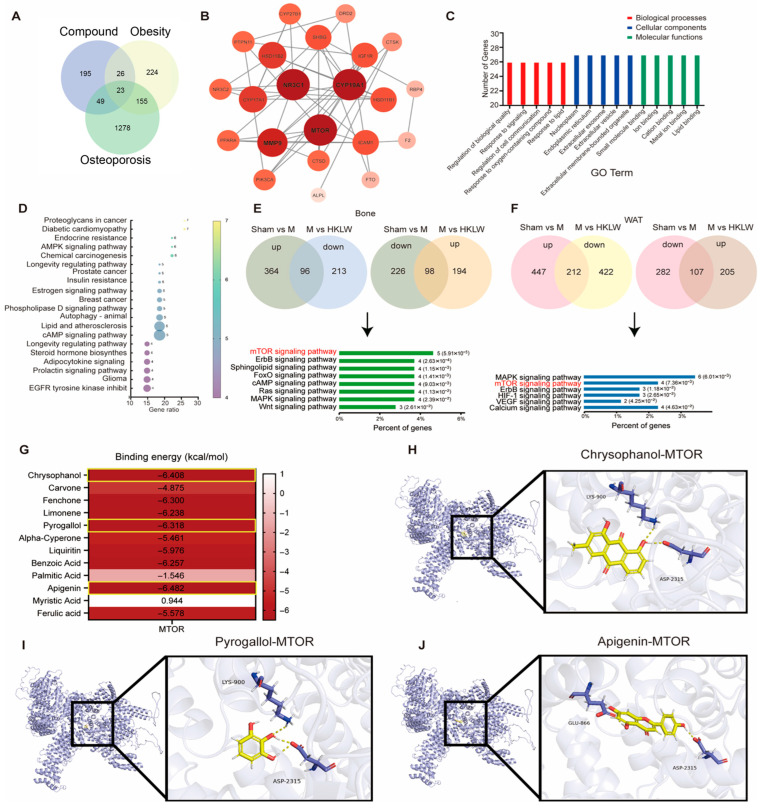
mTOR signaling mediates the therapeutic effects of KLW in mesenchymal stem cells. (**A**) Identification of potential therapeutic targets of KLW for osteoporosis and obesity. (**B**) Protein-Protein Interaction network of the KLW targeting osteoporosis and obesity. (**C**) Gene Ontology (GO) analysis of potential therapeutic targets. (**D**) KEGG analysis of potential therapeutic targets. (**E**) Quantitative proteomics of the mouse femur showing the number of altered proteins per group and enriched KEGG pathways. (**F**) Quantitative proteomics of the mouse white adipose tissue showing the number of altered proteins per group and enriched KEGG pathways. (**G**) Heatmap of binding energy between mTOR and compounds listed. (**H**–**J**) 3D Binding model analysis of mTOR protein (blue) with Chrysophanol, Pyrogallol and Apigenin (yellow). The key residues are shown sticks. H-bonds are shown as yellow dashed line.

Utilizing the SwissTargetPrediction platform, 293 high-confidence targets were identified for the 12 bioactive constituents of the herbal compound. Disease-specific targets for osteoporosis and obesity were systematically retrieved from GeneCards, OMIM, TTD, and PharmGKB. After deduplication and UniProt-based normalization, and applying a relevance score cutoff (>1.5, or >8.0 for high stringency), 1505 and 428 unique targets were obtained for osteoporosis and obesity, respectively. Intersection analysis with compound-predicted targets revealed 23 overlapping genes, proposed as putative therapeutic targets for KLW’s efficacy against both diseases (Figure 7A).

A protein–protein interaction (PPI) network of these 23 targets was constructed (Figure 7B), consisting of 23 nodes and 50 edges with an average node degree of 4.35. Based on degree centrality, the top 20 hub targets were identified as MTOR, CYP19A1, NR3C1, MMP9, CYP17A1, HSD11B2, SHBG, HSD11B1, IGF1R, ICAM1, CTSD, CYP27B1, NR3C2, PIK3CA, PTPN11, PPARA, CTSK, DRD2, FTO, and F2, with mTOR ranking highest in centrality (Table 1).

Gene Ontology analysis indicated significant enrichment in biological processes like the regulation of biological quality and responses to lipids and oxygen-containing compounds. Enriched molecular functions involved small molecule and ion binding, while key cellular components included the nucleoplasm, endoplasmic reticulum, and extracellular vesicles (Figure 7C). KEGG analysis identified nineteen significantly enriched pathways, linking metabolic dysregulation to obesity through pathways like adipocytokine signaling and insulin resistance, and to osteoporosis via estrogen and prolactin signaling. The enrichment of shared pathways such as steroid hormone biosynthesis highlighted common metabolic defects. Notably, the appearance of multiple cancer-related pathways implied a potential association between chronic metabolic disturbance and oncogenic risk (Figure 7D). Subsequently, to validate the aforementioned predictions, we performed proteomic analyses on the femoral bone and white adipose tissue of mice. In bone tissue, compared with the sham group, 364 proteins were up-regulated in the M group, among which 96 could be down-regulated by KLW. Meanwhile, 226 proteins were down-regulated in the M group, of which 98 could be up-regulated by KLW. Enrichment analysis of these 194 proteins revealed that the mTOR signaling pathway ranked as the most significantly enriched (Figure 7E). In adipose tissue, compared with the sham group, 447 proteins were up-regulated in the M group, with 212 of these being down-regulated by KLW. Additionally, 282 proteins were down-regulated in the M group, among which 107 could be up-regulated by KLW. Enrichment analysis of these 319 proteins indicated that the mTOR signaling pathway was the second most significantly enriched (Figure 7F). These findings collectively confirm our aforementioned predictions.

Molecular docking was employed to investigate binding interactions between mTOR and the compounds. Most compounds exhibited comparable binding energies, with Chrysophanol, Pyrogallol, and Apigenin showed the most favorable predicted binding energies (Figure 7G, Table 2). These compounds were predicted to bind within the active pockets of mTOR, represented as blue cartoons with compounds in yellow sticks and key residues as rods. Specifically, Chrysophanol formed three hydrogen bonds with LYS-900 and ASP-2315; Pyrogallol formed two with LYS-900 and ASP-2315; and Apigenin formed two with GLU-866 and ASP-2315 (Figure 7H–J). These results suggest that KLW components may modulate mTOR signaling in vivo.

### 2.11. Molecular Dynamics Simulations of mTOR-Ligand Binding in Model of Osteoporosis and Obesity

We employed molecular dynamics simulations to investigate the dynamic behavior of complexes formed between mTOR and three small-molecule ligands of KLW detected in the sera of treatment mice, namely Chrysophanol, Pyrogallol, and Apigenin. We performed molecular dynamics and measured key parameters including root mean square deviation (RMSD), root mean square fluctuation (RMSF), radius of gyration (Rg), solvent accessible surface area (SASA), number of hydrogen bonds, and energy contributions of hotspot residues were evaluated to assess the stability and conformational changes of the complexes.

**Figure 8:**
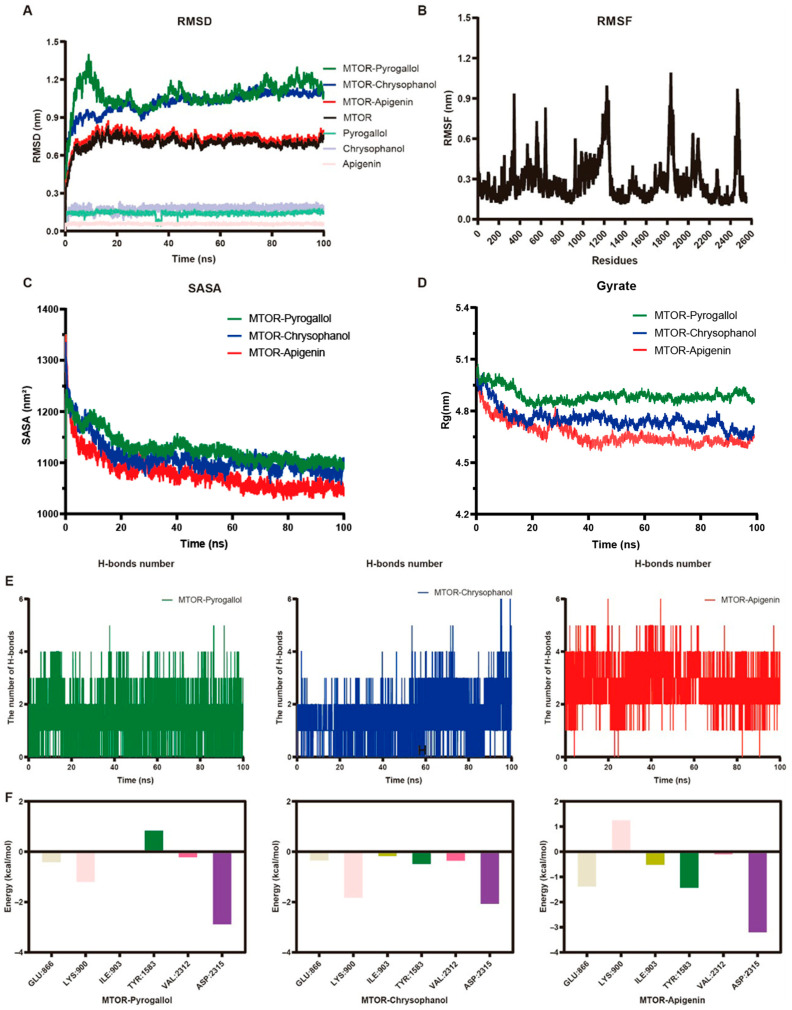
Molecular dynamics simulations of mTOR-Ligand binding in model of osteoporosis and obesity. (**A**) Root mean square deviation (RMSD) calculations of the protein and protein-ligand complexes over the simulation time. (**B**) Root mean square fluctuation (RMSF) measurements per residue, reflecting local flexibility of the protein backbone. (**C**) Solvent accessible surface area (SASA) variation throughout the trajectory. (**D**) Radius of gyration (Rg) studies indicating overall compactness and structural stability of the complexes. (**E**) Hydrogen bond analysis between the protein and ligands throughout the simulation trajectory. (**F**) Per-residue energy decomposition highlighting hot residues with significant contribution to binding free energy.

As shown, the RMSD plot (Figure 8A) illustrates the overall stability and conformational evolution of the docked complexes in comparison to the protein alone. Both the unliganded protein and the complexes exhibited initial fluctuations within the first ∼30 ns, after which the systems stabilized, indicating convergence under the simulation conditions. Throughout the simulation, the RMSD values of the complexes remained below 0.3 nm after equilibration. Comparative analysis revealed that the mTOR–Apigenin complex demonstrated superior stability relative to mTOR completes with Chrysophanol and Pyrogallol. The RMSF profile (Figure 8B) reflects the flexibility of residues within the receptor, with peaks indicating regions of high flexibility, likely corresponding to loop regions or areas near active sites that may participate in substrate binding. Enhanced flexibility in these regions may facilitate ligand interactions and functional adaptability, while lower RMSF values in other regions suggest structurally stable elements that maintained integrity throughout the simulation. We calculated SASA trajectories (Figure 8C) which describe the solvent exposure of each of the three complexes. Stable SASA values with minor fluctuations were observed, indicating consistent solvent accessibility throughout the simulation. This consistency suggests that ligand binding did not markedly alter the protein’s solvation pattern, which may be correlated with the preservation of functionality and stability in the bound state. Next, we developed Rg plots (Figure 8D) to evaluate the compactness of each of the three complexes. There, we found that each complex displayed stable Rg values, indicating that each complex maintained a consistent tertiary structure, with fluctuations within 0.2 nm after equilibration and no notable expansion or compaction, implying structural robustness. Minor variations are expected in molecular dynamics simulations and do not indicate significant unfolding or aggregation, further supporting the structural coherence of the complexes.

We performed hydrogen bond analysis (Figure 8E) which illustrates the dynamic variation in hydrogen bond interactions between the target protein and the small-molecule ligands throughout the simulation. Fluctuations in the number of hydrogen bonds indicate transient yet persistent interactions between the protein and the ligands. The stability of these interactions is critical, as hydrogen bonds play a key role in maintaining the integrity of the ligand–protein complex and may influence binding affinity and specificity. Finally, we performed energy decomposition analysis (Figure 8F) to identify key amino acid residues on mTOR with substantial energy contributions for binding, such as GLU:866, LYS:900, ILE:903, TYR:1583, VAL:2312, and ASP:2315.

The MM/GBSA binding free energy results are summarized in Table 3. The total binding free energies (ΔTotal) of the three ligands to mTOR indicate that Apigenin (−29.82 ± 1.82 kcal/mol) and Chrysophanol (−29.71 ± 1.56 kcal/mol) exhibit comparable binding affinities to mTOR, with both being superior to that of mTOR binding to Pyrogallol (−17.76 ± 1.82 kcal/mol). The strong binding of Apigenin is primarily attributed to a large favorable gas-phase energy (ΔGgas = −67.05) and a substantial electrostatic contribution (ΔEelec = −40.71), which, despite being partially offset by a polar solvation penalty (ΔEGB = +41.38), together with moderate van der Waals (ΔVDWAALS = −26.33) and nonpolar solvation (ΔEsurf = −4.15) terms, result in an optimal overall binding energy. In contrast, Chrysophanol shows a binding profile more dominated by nonpolar interactions (ΔVDWAALS = −29.28; ΔEsurf = −6.09), with moderate electrostatic contributions (ΔEelec = −14.63) and a corresponding polar desolvation penalty (ΔEGB = +40.30), ultimately yielding binding affinity nearly equivalent to that of Apigenin. Pyrogallol, however, demonstrated weaker van der Waals (−16.22) and electrostatic (−10.85) interactions, along with limited hydrophobic contributions (ΔEsurf = −1.39), leading to significantly reduced binding affinity compared to the other two ligands. Collectively, these results suggest that both Apigenin and Chrysophanol are promising mTOR-binding ligands, whereas binding by Pyrogallol may be of weaker affinity. Taken together, these molecular dynamics studies collectively indicate their stable binding to mTOR across all three complexes, with Apigenin demonstrating the most favorable profile.

### 2.12. KLW Treatment Inhibited mTOR Signaling in MSCs

**Figure 9:**
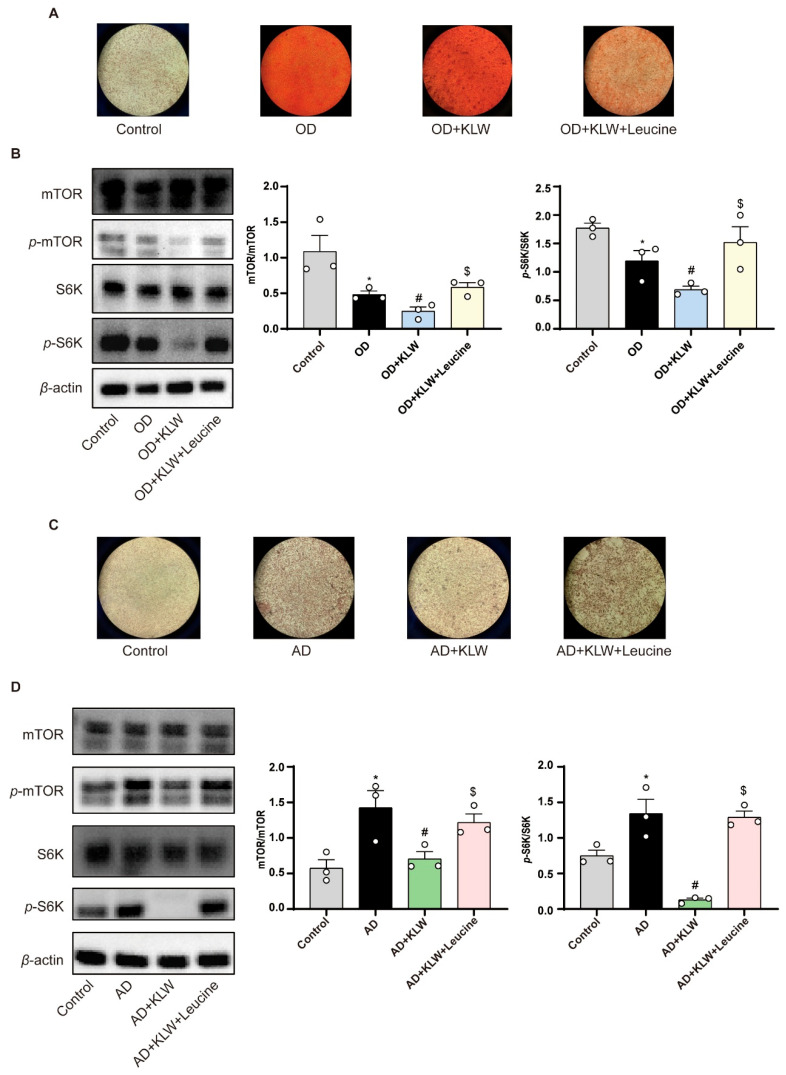
KLW treatment inhibited mTOR signaling in MSCs. (**A**) Alizarin Red staining of C3H10T1/2 cells. (**B**) Western blot analysis and quantification of mTOR, p-mTOR, S6K, and p-S6K protein expression during osteogenic differentiation. (**C**) Oil O Red staining of C3H10T1/2 cells. (**D**) Western blot analysis and quantification of mTOR, p-mTOR, S6K, and p-S6K protein expression during adipogenic differentiation. All data are presented as the mean ± SEM. n = 3. Statistical analysis was performed using one-way ANOVA followed by Tukey’s post hoc test: * *p* < 0.05 compared to the Control group, # *p* < 0.05 compared to the OD/AD group, $ *p* < 0.05 compared to the OD/AD + KLW group.

The mTOR protein is a 289 kDa serine-threonine kinase that integrates extracellular and intracellular signals, serving as a central regulator of cell metabolism, growth, proliferation, and survival [21]. Indeed, mTOR is capable of responding to estrogen signaling and plays a role in various cellular processes. Moreover, mTOR complex 1 (mTORC1) regulates the differentiation of MSCs, promoting adipogenesis by influencing the key regulator PPAR-γ, while inhibiting osteogenic differentiation through downstream S6K signaling [22]. Leucine supplementation has been found to exert an agonist effect on the mTOR pathway [23]. To investigate the mechanism by which KLW modulates mTOR signaling, we assessed the expression of key pathway proteins by Western blot. Total protein was extracted from C3H10T1/2 cells following a 3-day induction of osteogenic or adipogenic differentiation. To this end, we analyzed the levels of mTOR, phospho-mTOR (p-mTOR), S6K, and phospho-S6K (p-S6K) under these conditions. As shown, we found that leucine supplementation reversed the osteogenic-promoting and adipogenic-inhibiting effects mediated by KLW-containing serum (Figure 9A,C). KLW significantly suppressed the phosphorylation of mTOR and S6K during lineage differentiation. This suppression was effectively reversed by co-treatment with the mTOR agonist leucine, confirming that KLW acts through inhibition of the mTOR pathway (Figure 9B,D).

## 3. Discussion

In this study, we demonstrated that Kun-Ling Wan Formula (KLW), a classic herbal formula, could ameliorate osteoporosis and reduce fat accumulation in a preclinical model of osteoporosis in OVX mice fed a high-fat diet. Moreover, we found that the effects of KLW treatment were not associated with side-effects otherwise observed with estrogen-treatment. In addition, we found that the dual beneficial actions of KLW on bone and fat metabolism were associated with molecular markers of mTOR signal inhibition. Therefore, this study provides a pharmacological basis for the potential clinical use of KLW in osteoporosis as well as the putative underlying molecular mechanism through which it transduces its therapeutic effects on metabolic regulation and other pathological traits in this context.

The differential effects of KLW on adiposity and metabolic parameters under HFD and NCD conditions provide important mechanistic insights. While treatment with KLW could ameliorate osteoporosis in both HFD and NCD dietary regimens, its potent fat-reducing and metabolic-improving effects were evident only in HFD-fed OVX mice for the obvious reason that NCD does not induce fat production. This indicates that the anti-osteoporotic action of KLW is likely a direct action, possibly through a mechanism which targets the bone marrow microenvironment that is independent of adipose tissue signalling. In contrast, its systemic metabolic benefits may require a pre-existing, overtly dysmetabolic background—such as that created by HFD to become fully manifest. The HFD challenge may induce a state of widespread mTOR hyperactivation in peripheral metabolic tissues (e.g., liver, adipose tissue) [24] which, in turn, is effectively managed by KLW treatment through its capacity to suppress mTOR-inhibitory signalling. Consequently, KLW not only rectifies MSC fate determination in bone but also enhances whole-body lipid oxidation and energy expenditure, leading to a significant reduction in adipose tissue formation and improvements in glucose homeostasis. This context-dependent efficacy observed in our studies could be interpreted to explain that treatment with KLW normalizes aberrant mTOR signaling primarily under pathological conditions of osteoporosis in a preclinical model, and these results support the notion that KLW may be a potential new therapeutic strategy for managing the intertwined pathologies of osteoporosis and metabolic syndrome in postmenopausal women.

The dissociation between body weight and adiposity in HFD mice following KLW treatment is a noteworthy finding. While no significant change in total body weight was observed, a marked reduction in body fat percentage and absolute fat mass was accompanied by a concurrent increase in lean mass. This body composition shift can be attributed to the dual actions of KLW: it not only promoted lipolysis and inhibited adipogenesis, leading to fat loss, but also likely facilitated the preservation and/or accretion of lean tissue, potentially through improved systemic insulin sensitivity and energy expenditure. Consequently, the net effect on overall body weight remained neutral, underscoring the limitation of relying solely on body weight as an endpoint and highlighting the necessity of body composition analysis in metabolic studies.

A particularly intriguing finding of our study is the differential impact of KLW and estrogen on bone marrow adiposity. While both treatments effectively improved bone mass, only KLW treatment could significantly reduce the expansion of marrow adipose tissue induced by ovariectomy in both HFD and NCD. This discrepancy can be attributed to their distinct mechanisms of action. Estrogen primarily exerts its protective effect by suppressing osteoclastic bone resorption [25], with relatively modest direct effects on mesenchymal stem cell (MSC) differentiation. In contrast, KLW, through its bioactive components that we find likely targeting mTOR kinase through direct binding with mTOR, actively reprograms the fate determination of MSCs. By inhibiting mTORC1 signaling—a master regulator that promotes adipogenesis at the expense of osteogenesis [26]—KLW shifts the differentiation balance toward the osteoblastic lineage and away from the adipocytic lineage. This fundamental difference in molecular targeting may explain why KLW, unlike estrogen, achieves the dual benefit of increasing bone formation and concurrently reducing marrow fat accumulation.

In this study, KLW-containing serum was used for in vitro experiments, which is a common approach in the study of herbal formulas as it partially reflects the in vivo metabolic profile of the drug. However, we acknowledge the limitations of this method, including the complexity of the serum matrix, unclear concentrations of active components, and batch-to-batch variability. To address these limitations, we have analyzed the components of KLW-containing serum and identified potential active compounds. In future studies, we will use standardized extracts or purified active compounds at defined concentrations for in vitro validation, in order to more precisely elucidate the pharmacologically active basis and mechanism of action of KLW.

Based on our findings, KLW demonstrates a distinct and multi-faceted therapeutic profile that offers considerable advantages over both conventional estrogen replacement therapy as well as through single-component phytochemical interventions such as PSO or ASP. Firstly, KLW exhibits dual efficacy by simultaneously addressing bone loss and metabolic dysfunction. While estrogen effectively improved bone mineral density, it failed to reduce bone marrow adiposity in high-fat diet-fed OVX mice. Similarly, both PSO and ASP showed no significant effects on visceral fat reduction or glucose metabolism regulation. In contrast, KLW uniquely restored bone microarchitecture, reduced marrow and peripheral fat accumulation, and enhanced whole-body insulin sensitivity, indicating its capacity to co-regulate the “bone–fat–metabolism” axis. A key distinction between KLW and existing therapies lies in its dual efficacy across distinct metabolic states. This capacity to concurrently confer bone protection and metabolic improvement positions KLW as a promising therapeutic candidate for the growing population of patients with coexisting osteoporosis and obesity. Secondly, KLW presents a favorable safety profile attributable to its non-estrogenic mechanism. Notably, our study found that KLW administration did not increase uterine weight or elevate circulating estrogen levels, thereby circumventing the risks associated with estrogen-dependent pathways, such as breast hyperplasia, endometrial proliferation, and thromboembolic events.

A limitation of this study is the lack of absolute quantification for all detected serum components. Given that KLW comprises 31 herbal ingredients and yields a complex chemical profile, comprehensive quantification would require certified reference standards for each individual compound, many of which are not commercially available. Nevertheless, the consistent multi-batch fingerprint and the identification of key serum-available constituents provide a reliable basis for the interpretation of our biological findings. In addition, only a subset of proteomic findings, specifically the mTOR signaling axis, was subjected to in-depth experimental validation. While the proteomics screen served as a valuable discovery tool to nominate dysregulated pathways, comprehensive validation of all differentially expressed proteins was beyond the scope of this investigation. Future studies employing targeted proteomic approaches will be necessary to fully elucidate the broader proteomic landscape influenced by KLW.

The multi-target synergistic action of KLW underscores the strength of herbal formulae over single compounds. Unlike single-component treatment agents (in this study, PSO or ASP) that act through limited targets, KLW employs a combination of bioactive compounds (including apigenin, chrysophanol, and others confirmed in serum) that collectively modulate key signaling nodes, particularly the mTOR pathway, to influence osteogenesis, suppress adipogenesis, and improve metabolic homeostasis. This network-based mechanism aligns with the holistic philosophy of traditional medicine and provides a robust pharmacological foundation for its efficacy in complex conditions. KLW is effective to ameliorate the adipogenesis and osteoporosis features of a preclinical model of OVX + HFD mice, and that our findings raise the possibility that KLW might be an attractive therapy for postmenopausal osteoporosis through future clinical trials. Future investigations should focus on pharmacokinetic interactions among KLW’s multiple components and their translational potential in clinical settings.

## 4. Materials and Methods

### 4.1. Animals and Experimental Design

Female C57BL/6J mice (6–8 weeks old, weighing approximately 18–21 g) were obtained from the Experimental Animal Center of Peking University Health Science Center. All procedures were conducted in accordance with the Regulations for the Administration of Laboratory Animals issued by the Ministry of Health of the People’s Republic of China (Document No. 55, 2001). The animals were subjected to standard housing conditions and fed a high-fat diet (60% kcal from fat, D12492, HFD, Research Diets, New Brunswick, NJ, USA, provided by Beijing Boaopack Biotechnology Co., Ltd., Beijing, China). The experimental protocol was approved by the Animal Ethics Committee of Peking University Health Science Center (Approval No. LA2021134), and all procedures were performed in accordance with the guidelines of the Peking University Animal Research Committee. Healthy female C57BL/6J mice (6–8 weeks old) were randomly assigned to sham or bilateral ovariectomy (OVX) surgery groups. The OVX surgical procedures for mice were performed under aseptic conditions. Mice were anesthetized via intraperitoneal injection of a ketamine (100 mg/kg) and xylazine (10 mg/kg) mixture. Following confirmation of anesthesia for each mouse, the hair on their dorsal lumbar area was shaved and disinfected. A single midline incision (approximately 1–1.5 cm) was made in the skin over the lumbar spine. For the OVX group, for each ovary, a small opening was made in the underlying muscle wall just lateral to the midline. The ovarian fat pad, which contains the ovary, was gently exteriorized using forceps. The ovary was identified, and the uterine horn was ligated with sterile suture below the oviduct and ovary. The ovary was then removed by excision above the ligation. The uterine horn was returned into the abdominal cavity, and the muscle wall was closed with a single suture. The same procedure was repeated on the contralateral side. In sham-operated mice, an identical protocol was followed, from anesthesia to midline skin incision and the exteriorization of the ovarian fat pads; however, the ovaries were not ligated or excised and were returned intact into the abdominal cavity. For all animals, the skin incision was finally closed with wound clips.

All OVX mice were weighed, and their body weights were used to perform stratified randomization using a computer-generated random number sequence (Microsoft Excel RAND function). This ensured an even distribution of baseline body weight across all experimental groups prior to the initiation of treatment. OVX mice were further divided into six treatment groups, namely Sham (drinking water), Model (M, drinking water), Estradiol (E_2_, 1 mg/kg/day), Psoralen (PSO, 20 mg/kg/day), Asperosaponin VI (ASP, 20 mg/kg/day), Low-dose KLW (LKLW, 1.17 g/kg/day) and High-dose KLW (HKLW, 2.34 g/kg/day). Kun-Ling Wan Formula (KLW) was supplied by Tasly Pharmaceutical Group Co., Ltd. (Tianjin, China; batch no. 20180843). The clinical dosage of KLW in adults is defined as 0.13 g/kg/day. Thus, an equivalent murine dose was defined as 1.17 g/kg/day according to established interspecies dose conversion criteria [27]. An additional high-dosage group received 2.34 g/kg/day, representing twice the clinical equivalence, to investigate dose-responsive therapeutic efficacy. Psoralen (PSO) and asperosaponin VI (ASP), each with a purity exceeding 98%, were procured from Bailensi Co., Ltd. (Chengdu, China). These compounds were selected as positive controls based on their well-documented efficacy in enhancing osteogenic activity and attenuating bone loss in established preclinical models of postmenopausal osteoporosis. β-Estradiol was obtained from Sigma-Aldrich (St. Louis, MO, USA). All mice were fed a high-fat diet and treated via daily oral gavage for 28 days. During the outcome assessment phase, the investigators responsible for collecting and weighing tissue were blinded to the group allocation. Specifically, animals were identified only by cage numbers and ear tags, with the corresponding treatment codes kept in a sealed document by a separate lab member not involved in the tissue harvest or data analysis.

### 4.2. microCT Analysis

The femurs of mice were carefully isolated, ensuring the preservation of the femoral head, and excess muscle tissue was removed. The femurs were then fixed in 10% formalin for 48 h. After fixation, the femurs were placed on a specimen holder and scanned using a microCT scanner (Quantum FX, PerkinElmer, Waltham, MA, USA). Structural images of the trabeculae in the femoral head were obtained, along with relevant trabecular bone data.

### 4.3. MRI Analysis

Mice were numbered and anesthetized using isoflurane via a Midmark anesthesia machine (Midmark, Versailles, OH, USA). For body composition analysis using nuclear magnetic resonance (NMR), mice were sequentially numbered and weighed, and then individually placed into the NMR machine (TRIO, Siemens, Munich, Germany). The corresponding body weight readings for each mouse were then entered into the system prior to measurement. The NMR analysis provided data on fat mass and lean mass, which were statistically analyzed to calculate the ratios of fat mass to body weight and lean mass to body weight, respectively. This method allowed for precise and non-invasive quantification of body composition in the treated mice.

For femurs fat analysis, the lower body of each mouse was scanned with a medium coil in the MRI scanner (TRIO, Siemens, Munich, Germany). The percentages of fat content in the bone marrow of both femurs were assessed, and the average values were calculated.

### 4.4. Oral Glucose Tolerance Tests (OGTT) and Insulin Tolerance Tests (ITT)

For oral glucose tolerance tests (OGTT), mice were fasted for 16–18 h overnight prior to sampling. A glucose solution was prepared by dissolving 5 g of glucose in 1 mL of physiological saline, which was then boiled and diluted to a final volume of 5 mL. The glucose solution was administered via oral gavage at a dose of 3 g glucose per kg body weight, calculated as 3 µL × body weight (g). Blood glucose levels were measured at 0, 15, 30, 60, 90, and 120 min post-administration. For insulin tolerance tests (ITT), mice were fasted for 6 h during the day and 4 h at night prior to sampling. Insulin was diluted in physiological saline to a concentration of 1/3 U/mL and administered via intraperitoneal injection at a dose of 3 µL × body weight (g). Blood glucose levels were recorded at 0, 15, 30, 60, 90, and 120 min following insulin injection. Both tests were conducted to evaluate glucose metabolism and insulin sensitivity in treated mice.

### 4.5. Metabolic Cage Experiments

Prior to the experiment, mice were acclimatized to metabolic cages for a minimum of 48 h to minimize stress-induced variability in metabolic parameter readings, with ad libitum access to food and water provided throughout this period. Metabolic cages (CLAMS, Columbus Instruments, Columbus, OH, USA) were calibrated according to the manufacturer’s instructions to ensure accurate measurement of oxygen consumption (VO_2_), carbon dioxide production (VCO_2_), food intake, water consumption, and locomotor activity, with the cages maintained in a controlled environment featuring a 12-h light/dark cycle, constant temperature (22 ± 1 °C), and humidity (50 ± 10%). Following acclimatization, baseline metabolic parameters were recorded over a 24-h period to establish individual reference values for each mouse, including VO_2_, VCO_2_, respiratory exchange ratio (RER), energy expenditure, food and water intake, and spontaneous activity.

### 4.6. HE Staining

Tissue samples including mouse femurs and adipose tissue were fixed in 4% paraformaldehyde for 24–48 h, followed by decalcification of bone specimens in EDTA solution for 4 weeks with needle-puncture verification of complete decalcification; all tissues were processed through graded ethanol dehydration, xylene clearance, and paraffin embedding using standard histological protocols (Leica EG1150H embedding station, Leica Biosystems, Nussloch, Germany); serial sections were cut at 5 μm thickness using a rotary microtome (Leica RM2235, Leica Biosystems, Nussloch, Germany), mounted on poly-lysine coated slides. Tissue sections were dewaxed through three changes of xylene (10 min each) and rehydrated in a graded ethanol series (100%, 95%, 85%, 70%—3 min each) followed by a 5-min rinse in distilled water. Nuclei were stained with Mayer’s hematoxylin solution (Sigma-Aldrich, St. Louis, MO, USA) for 8 min at room temperature, followed by washing in running tap water for 10 min until sections turned blue. Cytoplasmic staining was performed using eosin Y solution (0.5% in distilled water) for 3 min. Sections were then dehydrated through graded alcohols (70%, 85%, 95%, 100%—1 min each) and cleared in three changes in xylene (3 min each). Finally, slides were mounted with neutral balsam (Sigma-Aldrich) under cover slips and air-dried for 24 h before microscopic examination. All staining procedures were performed at room temperature with gentle agitation, and reagents were freshly prepared every two weeks to ensure consistent staining quality.

### 4.7. MSC Differentiation

The C3H10T1/2 mouse embryonic mesenchymal stem cell line was purchased from Zhongqiao Xinzhou (Shanghai, China). Cells were cultured in DMEM supplemented with 10% fetal bovine serum (FBS), 100 U/mL penicillin, and 100 U/mL streptomycin, with the culture medium pre-warmed at 37 °C for 30 min before use. For adipogenic differentiation, the culture medium was supplemented with 1 μg/mL insulin, 1 μmol/L dexamethasone, and 0.5 mmol/L 3-isobutyl-1-methylxanthine (IBMX). For osteogenic differentiation, the medium was supplemented with 0.1 μmol/L dexamethasone, 10 mmol/L β-glycerophosphate disodium, 2.5 mmol/L calcium chloride, and 10^−8^ mol/L vitamin D. Osteogenic differentiation experiments or cell passaging procedures were performed when cultured cells reached 70–80% confluency, whereas adipogenic differentiation was initiated once the cells reached 100% confluency.

### 4.8. Oil Red O Staining

Cells were cultured in wells containing coverslips until collection. On the day of the experiment adherent cells on such coverslips within each well of culture plates were gently washed three times with PBS. The cells were then fixed with 4% paraformaldehyde for 30 min, followed by three washes with PBS. An oil red O stock solution was diluted to a working solution through a 3:2 ratio with deionized water and filtered through filter paper for use. Cells were stained with the working oil red O solution for approximately 30 min, ensuring that the solution completely covered the bottom of the plate. Excess dye was removed by rinsing with 75% ethanol, followed by PBS washing. The samples were then mounted with glycerol gelatin and observed under a microscope.

### 4.9. Alizarin Red Staining

Adherent cells cultured on coverslips within culture plates were gently washed three times with PBS, followed by fixation in 4% paraformaldehyde for 30 min. The samples were then washed three times with PBS. After staining with Alizarin Red solution for 5 min, the cells were washed three times with PBS. Calcium deposition-positive cells were observed under a microscope, displaying an orange-red color.

### 4.10. Western Blot

Protein extraction and Western blot analysis were both performed as follows: mouse femurs were flushed with RIPA lysis buffer (Thermo Fisher, #89900, Waltham, MA, USA) containing protease and phosphatase inhibitors (Roche, #04906845001, Basel, Switzerland) to collect bone marrow cells, while adherent cells were directly lysed in culture plates; total protein concentration was determined using BCA assay (Pierce, #23225, Waltham, MA, USA) with bovine serum albumin as standard. For each lysate preparation, 30 μg of protein was prepared and separated using a 4–12% Bis-Tris gradient gel (Invitrogen, #NP0335BOX, Carlsbad, CA, USA) and transferred on to a PVDF membrane (Millipore, #IPFL00010, Billerica, MA, USA) through a wet transfer system (electrophoresis at 100 V for 90 min). Subsequently, membranes were blocked with 5% BSA in TBST for 1 h at room temperature and incubated overnight at 4 °C with primary antibodies against mTOR (Cell Signaling, #2983, 1:1000, Danvers, MA, USA), p-mTOR (Ser2448, Cell Signaling, #5536, 1:800), S6K (Cell Signaling, #9202, 1:1000), and p-S6K (Thr389, Cell Signaling, #9234, 1:800). The next day, three washes with TBST were performed on the PVDF membranes, followed by incubation with HRP-conjugated secondary antibodies (Jackson ImmunoResearch, #115-035-003, 1:5000, West Grove, PA, USA) for 1 h at room temperature. Immunodetected protein bands were visualized using ECL substrate (Bio-Rad, #1705060, Hercules, CA, USA) and quantified by ImageLab software (version 6.1) with immunoblotted signals for β-actin (Cell Signaling, #4967, 1:5000, Danvers, MA, USA) used as loading controls.

### 4.11. Quantitative Polymerase Chain Reaction (QPCR)

Total RNA was extracted from mouse femurs and cultured cells using TRIzol reagent (Invitrogen, #15596026, Carlsbad, CA, USA) according to the manufacturer’s protocol. Bone marrow was flushed with TRIzol using a 21-gauge needle and homogenized using a rotor-stator homogenizer (IKA T10 basic) at 20,000 rpm for 30 s while cultured cells were directly lysed in TRIzol. RNA concentration and purity were both determined using a NanoDrop spectrophotometer (Thermo Scientific, Waltham, MA, USA) with all samples confirmed to feature A260/A280 ratios of between 1.8 and 2.0. Next, 1 μg of total RNA from each sample was reverse transcribed into cDNA using PrimeScript RT Master Mix (Takara, #RR036A, Kusatsu, Japan) with the following cycling conditions: 37 °C for 15 min and 85 °C for 5 s; quantitative PCR was performed using TB Green Premix Ex Taq II (Takara, #RR820A, Kusatsu, Japan) on a QuantStudio 6 Flex system (Applied Biosystems, Waltham, MA, USA) with the following parameters: initial denaturation at 95 °C for 30 s, followed by 40 cycles of 95 °C for 5 s and 60 °C for 30 s; all reactions were run in triplicate with melt curve analysis to confirm amplification specificity, and relative gene expression was calculated using the 2^(−ΔΔCt)^ method with signals for β-actin QPCR as an endogenous control. Primer sequences used in this study are provided in Supplementary Table S2.

### 4.12. Preparation of Drug-Containing Serum

Sprague-Dawley (SD) rats weighing 160–180 g were administered KLW via oral gavage at a dose of 1.82 g/kg for three consecutive days, ensuring consistent dosing time each day. One hour after the final gavage, blood was collected from the abdominal aorta of each mouse. After allowing the blood samples to coagulate at room temperature, these were centrifuged at 1000× *g* for 20 min at 4 °C. The supernatant serum was then collected for each sample, aliquoted, and stored at −20 °C. Before use, the serum was filtered through a cell strainer.

### 4.13. Mass Spectrometric Identification of Active Constituents in the Sera of Mice

Serum samples of control and treated mice were prepared by protein precipitation with ice-cold acetonitrile (1:3 serum:acetonitrile ratio) followed by centrifugation at 14,000× *g* for 15 min at 4 °C. The supernatant was then transferred to autosampler vials and analyzed using an UHPLC-Q-TOF/MS system (Agilent 1290 Infinity II/6550 iFunnel, Santa Clara, CA, USA) with a ZORBAX Eclipse Plus C18 column (2.1 × 100 mm, 1.8 μm) maintained at 40 °C. Chromatographic separation was achieved using a binary gradient of 0.1% formic acid in water (mobile phase A) and 0.1% formic acid in acetonitrile (mobile phase B) at a flow rate of 0.3 mL/min with the following elution program: 0–2 min 5% B, 2–20 min 5–95% B, 20–25 min 95% B, 25–25.1 min 95% to 5% B, 25.1–30 min 5% B. MS detection was performed in both positive and negative ionization modes with the following parameters: gas temperature 250 °C, drying gas 12 L/min, nebulizer 35 psi, sheath gas temperature 350 °C, sheath gas flow 11 L/min, fragmentor voltage 175 V, and mass range *m*/*z* 100–1700. Reference mass correction was applied using reference ions at *m*/*z* 121.0509 and 922.0098 in positive mode and *m*/*z* 112.9856 and 966.0007 in negative mode. Data acquisition and analyses were performed using MassHunter Workstation Software (version B.10.0) with targeted screening for known KLW components and untargeted profiling for potential metabolites.

### 4.14. Target Predictions for KLW Components

Putative protein targets of KLW were predicted using the Swiss Target Prediction platform http://www.swisstargetprediction.ch (accessed on 20 October 2025). This tool employs both two-dimensional and three-dimensional chemical similarity assessments to identify the most probable protein targets of small molecules, with prediction confidence rankings based on a proprietary scoring algorithm [28]. To ensure comprehensive target coverage while maintaining prediction reliability, all predicted targets with a probability score > 0 were retained for initial analysis.

### 4.15. Therapeutic Targets for Osteoporosis and Obesity

For gene expression analyses, disease-associated genes related to osteoporosis and obesity were retrieved from public databases including DisGeNET http://www.disgenet.org/web/DisGeNET/menu/home (accessed on 27 October 2025), GeneCards https://www.genecards.org (accessed on 27 October 2025), the Gene Expression Omnibus (GEO), and the Comparative Toxicogenomics Database, CTD; http://ctdbase.org/ (accessed on 27 October 2025).

### 4.16. Protein–Protein Interaction (PPI) Network

A protein–protein interaction (PPI) network was constructed using the STRING database https://string-db.org/ (accessed on 27 October 2025) based on the predicted therapeutic targets of the compound formulation for osteoporosis and obesity [29]. The minimum required interaction score was set to “medium confidence” (0.400) to ensure reliable interactions. A PPI enrichment *p*-value threshold of <1.0 × 10^−6^ was applied. Subsequently, the resulting network was imported into Cytoscape (version 3.10), and the CytoNCA plugin was used to compute the degree centrality (DC) of each node [30]. To identify core therapeutic targets in an unbiased manner, hub targets were defined as nodes with a DC value ≥ twofold the median DC value of all nodes in the PPI network. This threshold-based approach has been widely adopted in network pharmacology studies to objectively prioritize biologically significant targets. Final visualization of the PPI network was performed using Cytoscape software (version 3.10) [31].

### 4.17. Functional Annotation and Pathway Enrichment Analysis

Gene Ontology (GO) enrichment analysis was conducted using the “clusterProfiler” R package, covering three functional categories: GOTERM_BP_DIRECT (biological process), GOTERM_CC_DIRECT (cellular component), and GOTERM_MF_DIRECT (molecular function). Kyoto Encyclopedia of Genes and Genomes (KEGG) pathway enrichment and disease association analyses were performed using the DAVID database [24].

### 4.18. Molecular Docking

Molecular docking studies were performed using the Glide module (Schrödinger Suite 2022-1, Schrödinger, LLC, New York, NY, USA) with the following protocol: the crystal structure of mTOR kinase domain (PDB ID: 4JT6) was prepared through Protein Preparation Wizard involving hydrogen addition, bond order assignment, removal of crystallographic water molecules beyond 5 Å from the ligand, and optimization of protonation states at pH 7.0 ± 2.0; the co-crystallized ligand was used to define the centroid of the active site grid box (15 × 15 × 15 Å). The small molecule compound was prepared using LigPrep module with OPLS4 force field to generate possible ionization states and stereoisomers at pH 7.0 ± 2.0. Docking calculations were executed in standard precision (SP) mode with flexible ligand sampling and enhanced van der Waals scaling for nonpolar atoms. Resultant poses were ranked according to Glide docking score and visual inspection was conducted using Maestro molecular visualization platform to analyze key hydrogen bonding and hydrophobic interactions within the phosphorylation pocket.

### 4.19. Molecular Dynamics Simulations

Gromacs2022.3 software (version 2022.3) was used for molecular dynamics simulation studies. For small molecule preprocessing, AmberTools22 (version 22) is used to add GAFF force-fields to small molecules, while Gaussian 16 W is used to hydrogenate small molecules and to calculate RESP potential. The simulation conditions were carried out at a static temperature of 300 K and atmospheric pressure (1 Bar). Amber99sb-ildn was used as force field features, while water molecules were used as the solvent (Tip3p water model), and the total charge of the simulation system was neutralized by adding 3 Na+ ions. The simulation system adopts the steepest descent method to minimize the energy, and then carries out the isothermal isovolumic ensemble (NVT) equilibrium and isothermal isobaric ensemble (NPT) equilibrium for 100,000 steps, respectively, with the coupling constant of 0.1 ps and the duration of 100 ps. Finally, free molecular dynamics simulations were performed. The process consisted of 5,000,000 steps, the step length was 2fs, and the total duration was 100 ns. After each simulation was completed, the built-in tool of the software was used to analyze the trajectory, and the root-mean-square variance (RMSD), root-mean-square fluctuation (RMSF) and protein rotation radius of each amino acid trajectory were all calculated, combined with the free energy topography and other data features.

### 4.20. Statistical Analyses

Statistical analyses were performed using GraphPad Prism 9.0 with data expressed as the mean ± SEM. Each data point (circle) represents an individual animal, and the number of circles per group indicates the sample size (n) for that group. Comparisons among multiple groups were conducted using one-way ANOVA followed by Tukey’s multiple comparisons test. No data were excluded from the data analysis. Significance was set at *p* < 0.05.

## 5. Conclusions

In conclusion, KLW alleviates osteoporosis and fat accumulation in a preclinical model of osteoporosis in ovariectomized mice fed a high-fat diet, likely through a cellular mechanism that involves enhancing osteogenesis and inhibiting adipogenesis of MSCs through mTOR pathway regulation. These findings provide a scientific foundation for its future development as a therapeutic for managing osteoporosis and related metabolic disorders.

## Figures and Tables

**Table 1:** Top 20 targets by degree in the PPI network for treating osteoporosis and obesity.

Name	Degree	Betweenness Centrality	Closeness Centrality
MTOR	8	0.119654	0.552632
CYP19A1	8	0.154734	0.538462
NR3C1	8	0.219365	0.552632
MMP9	7	0.158466	0.552632
CYP17A1	6	0.046958	0.466667
HSD11B2	6	0.016585	0.477273
SHBG	5	0.163424	0.5
HSD11B1	5	0.013808	0.466667
IGF1R	5	0.026321	0.5
ICAM1	5	0.089484	0.512195
CTSD	4	0.077559	0.477273
CYP27B1	4	0.008649	0.456522
NR3C2	4	0.005257	0.428571
PIK3CA	4	0.009172	0.456522
PTPN11	4	0.024858	0.42
PPARA	4	0.002976	0.488372
CTSK	3	0.095238	0.411765
DRD2	3	0.050886	0.446809
FTO	2	0.015019	0.388889
F2	2	0.020238	0.381818

MTOR, mechanistic target of rapamycin; CYP19A1, cytochrome P450 family 19 subfamily A member 1; NR3C1, nuclear receptor subfamily 3 group C member 1; MMP9, matrix metallopeptidase 9; CYP17A1, cytochrome P450 family 17 subfamily A member 1; HSD11B2, hydroxysteroid 11-beta dehydrogenase 2; SHBG, sex hormone-binding globulin; HSD11B1, hydroxysteroid 11-beta dehydrogenase 1; IGF1R, insulin-like growth factor 1 receptor; ICAM1, intercellular adhesion molecule 1; CTSD, cathepsin D; CYP27B1, cytochrome P450 family 27 subfamily B member 1; NR3C2, nuclear receptor subfamily 3 group C member 2; PIK3CA, phosphatidylinositol-4,5-bisphosphate 3-kinase catalytic subunit alpha; PTPN11, protein tyrosine phosphatase non-receptor type 11; PPARA, peroxisome proliferator-activated receptor alpha; CTSK, cathepsin K; DRD2, dopamine receptor D2; FTO, fat mass and obesity-associated protein; F2, coagulation factor II.

**Table 2:** Binding energy of protein with compounds.

Compounds ID	Binding Energy (kcal/mol)
Chrysophanol	−6.408
Carvone	−4.875
Fenchone	−6.300
Limonene	−6.238
Pyrogallol	−6.318
Alpha-Cyperone	−5.461
Liquiritin	−5.976
Benzoic Acid	−6.257
Palmitic Acid	−1.546
Apigenin	−6.482
Myristic Acid	−5.230
Ferulic acid	−5.578

**Table 3:** Molecular mechanics with generalized born and surface area.

Contribution Components	MTOR-Chrysophanol	MTOR-Pyrogallol	MTOR-Apigenin
Δ_VDWAALS_	−29.28 ± 0.78	−16.22 ± 0.31	−26.33 ± 0.32
ΔE_elec_	−14.63 ± 1.39	−10.85 ± 1.68	−40.71 ± 1.53
ΔE_GB_	40.30 ± 1.95	20.70 ± 0.60	41.38 ± 0.93
ΔE_surf_	−6.09 ± 0.32	−1.39 ± 0.09	−4.15 ± 0.05
ΔG_gas_	−63.91 ± 1.51	−47.07 ± 1.71	−67.05 ± 1.56
ΔG_solvation_	34.21 ± 1.96	14.31 ± 0.60	37.22 ± 0.93
ΔTotal	−29.71 ± 1.56	−17.76 ± 1.82	−29.82 ± 1.82

Δ_VDWAAALS_, van der Waals and nonpolar solvation energy; Δ_Eelec_, electrostatic energy; ΔE_GB_, polar solvation energy calculated using the Generalized Born model; ΔE_surf_, nonpolar solvation energy; ΔG_gas_, total gas phase energy; ΔG_solution_, total solvation energy; ΔTotal, total binding free energy. All values are presented as mean ± standard deviation (kcal/mol).

## Data Availability

The original contributions presented in this study are included in the article/Supplementary Material. Further inquiries can be directed to the corresponding authors.

## Supplementary Materials

The following supporting information can be downloaded at: https://www.mdpi.com/article/10.3390/ph19050719/s1, Figure S1: KLW alleviated osteoporosis in OVX mice fed a normal chow diet. (**A**) Common peak matching profile of 11 batches of KLW. (**B**) Alanine aminotransferase (ALT). (**C**) Aspartate aminotransferase (AST). (**D**) creatinine (CREA), and (**E**) uric acid (UA) were measured in each group. (**F**) Uterine blotted weight. Mice were randomly assigned to undergo either sham surgery or ovariectomy (OVX). After a 3-day postoperative recovery period, all animals were initiated on a normal-chow diet (NCD) which was maintained throughout the experimental period. Following diet initiation, mice received daily gavage treatments for 28 consecutive days according to their respective group assignments. The Sham, M, E2, PSO, ASP, LKLW, and HKLW groups represent the sham-operated group, model group, estrogen group, psoralen group, asperosaponin VI group, low-dose KLW group, and high-dose KLW group, respectively. Uterine blotted weight. (**G**) Schematic diagram of animal grouping and drug administration regimen. (**H**–**N**) Quantitative analysis of bone microarchitectural parameters from micro-CT: (**H**) bone mineral density (BMD), (**I**) bone volume fraction (BV/TV), (**J**) trabecular separation (Tb.Sp), (**K**) trabecular number (Tb.N), (**L**) trabecular bone density (Tb.BMD), (**M**) trabecular thickness (Tb.Th), and (**N**) structure model index (SMI). All data are presented as the mean ± SEM. Statistical analysis was performed using one-way ANOVA followed by Tukey’s post hoc test: $ *p* < 0.05 compared to the NCD-Sham group, % *p* < 0.05 compared to the NCD-M group; Figure S2: KLW does not significantly affect body weight in HFD-fed mice. Body weight was measured at each time point, and the area under the curve (AUC) was calculated for statistical analysis. (**A**) Body weight curve. (**B**) Area under the curve. All data are presented as the mean ± SEM. Statistical analysis was performed using one-way ANOVA followed by Tukey’s post hoc test; Figure S3: KLW does not alter body weight or body composition in ovariectomized mice under NCD. Body weight was measured at each time point, and the area under the curve (AUC) was calculated for statistical analysis. (**A**) Body weight curve. (**B**) Area under the curve. (**C**) Body fat percentage. (**D**) Lean mass percentage. (**E**) uterine white adipose tissue weight. (**F**) Renal white adipose tissue weight. (**G**) Volume of abdominal adipose tissue. (**H**) Volume of subcutaneous adipose tissue. All data are presented as the mean ± SEM. Statistical analysis was performed using one-way ANOVA followed by Tukey’s post hoc test; Figure S4: KLW treatment resulted in a reduction in bone marrow fat content in OVX mice under NCD. Statistical results of the proton density fat fraction (PDFF) in the femoral bone marrow of mice. The values represent the average of bilateral measurements. All data are presented as the mean ± SEM. Statistical analysis was performed using one-way ANOVA followed by Tukey’s post hoc test: $ *p* < 0.05 compared to the NCD-Sham group, % *p* < 0.05 compared to the NCD-M group; Figure S5: Effects of KLW on metabolic homeostasis in NCD mice. (**A**,**B**) Oral Glucose Tolerance Test (OGTT) and AUC. (**C**,**D**) Insulin Tolerance Test, (ITT) and AUC. (**E**,**F**) Energy expenditure curve and AUC. (**G**,**H**) Activity curve and AUC. (**I**,**J**) Respiratory Exchange Ratio (RER) and AUC. All data are presented as the mean ± SEM. Statistical analysis was performed using one-way ANOVA followed by Tukey’s post hoc test: $ *p* < 0.05 compared to the NCD-Sham group, % *p* < 0.05 compared to the NCD-M group; Table S1: Composition of KLW; Table S2: Primers; Video S1: Three-dimensional micro-CT reconstructions of the proximal femoral metaphysis of Sham group mice; Video S2: Three-dimensional micro-CT reconstructions of the proximal femoral metaphysis of M group mice; Video S3: Three-dimensional micro-CT reconstructions of the proximal femoral metaphysis of E_2_ group mice; Video S4: Three-dimensional micro-CT reconstructions of the proximal femoral metaphysis of PSO group mice; Video S5: Three-dimensional micro-CT reconstructions of the proximal femoral metaphysis of ASP group mice; Video S6: Three-dimensional micro-CT reconstructions of the proximal femoral metaphysis of LKLW group mice; Video S7: Three-dimensional micro-CT reconstructions of the proximal femoral metaphysis of HKLW group mice.

## Abbreviations

The following abbreviations are used in this manuscript:

ASP	Asperosaponin VI
BMD	Bone Mineral Density
BV/TV	Bone Volume/Total Volume
E_2_	Estradiol
GO	Gene Ontology
H&E	Hematoxylin and Eosin
HFD	High-Fat Diet
HKLW	High-dose Kun-Ling Wan Formula
ITT	Insulin Tolerance Test
KEGG	Kyoto Encyclopedia of Genes and Genomes
KLW	Kun-Ling Wan Formula
LKLW	Low-dose Kun-Ling Wan Formula
M	Model
MRI	Magnetic Resonance Imaging
mTOR	Mammalian Target of Rapamycin
MSC	Mesenchymal Stem Cell
NCD	Normal Chow Diet
OGTT	Oral Glucose Tolerance Test
OVX	Ovariectomized
PDFF	Proton Density Fat Fraction
PPI	Protein-Protein Interaction
PSO	Psoralen
RER	Respiratory Exchange Ratio
Rg	Radius of Gyration
RMSD	Root Mean Square Deviation
RMSF	Root Mean Square Fluctuation
SASA	Solvent Accessible Surface Area
SMI	Structure Model Index
Tb.BMD	Trabecular Bone Mineral Density
Tb.N	Trabecular Number
Tb.Sp	Trabecular Separation
Tb.Th	Trabecular Thickness
VCO_2_	Carbon Dioxide Production
VO_2_	Oxygen Consumption
