# Supplementary material for: Kun-Ling Wan Formula Ameliorates Postmenopausal Osteoporosis and Adipose Accumulation by Suppressing mTOR Signaling in Mesenchymal Stem Cells
Source: Pharmaceuticals (Basel). 2026 Apr 30;19(5):719. doi: 10.3390/ph19050719 (PMC13210056; doi:10.3390/ph19050719)
Supplement: Supplementary file 1 [file pharmaceuticals-19-00719-s001.zip › proofreading_version_Supplementary_Pharmaceuticals_20260428.pdf]

Supplementary Materials for

Kun-Ling Wan Formula Ameliorates Postmenopausal Osteo-porosis and Adipose Accumulation by Suppressing mTOR Signaling in Mesenchymal Stem Cells

Xiaoqing Lu, Tingting Xie, He Lan, Yaqi Fan, Jie Yang, Qianzan Liao, Yuxin Jin, Yaoxuan Zhu, Jingxin Zhang, Dexin Li, Chunshui Pan, Quan Li, Kai Sun, Xinmei Huo, Ting Yuwen, Jing-Yan Han *, Yin Li *.

Correspondence to: yinli@bjmu.edu.cn, hanjingyan@bjmu.edu.cn

Figure. S1.

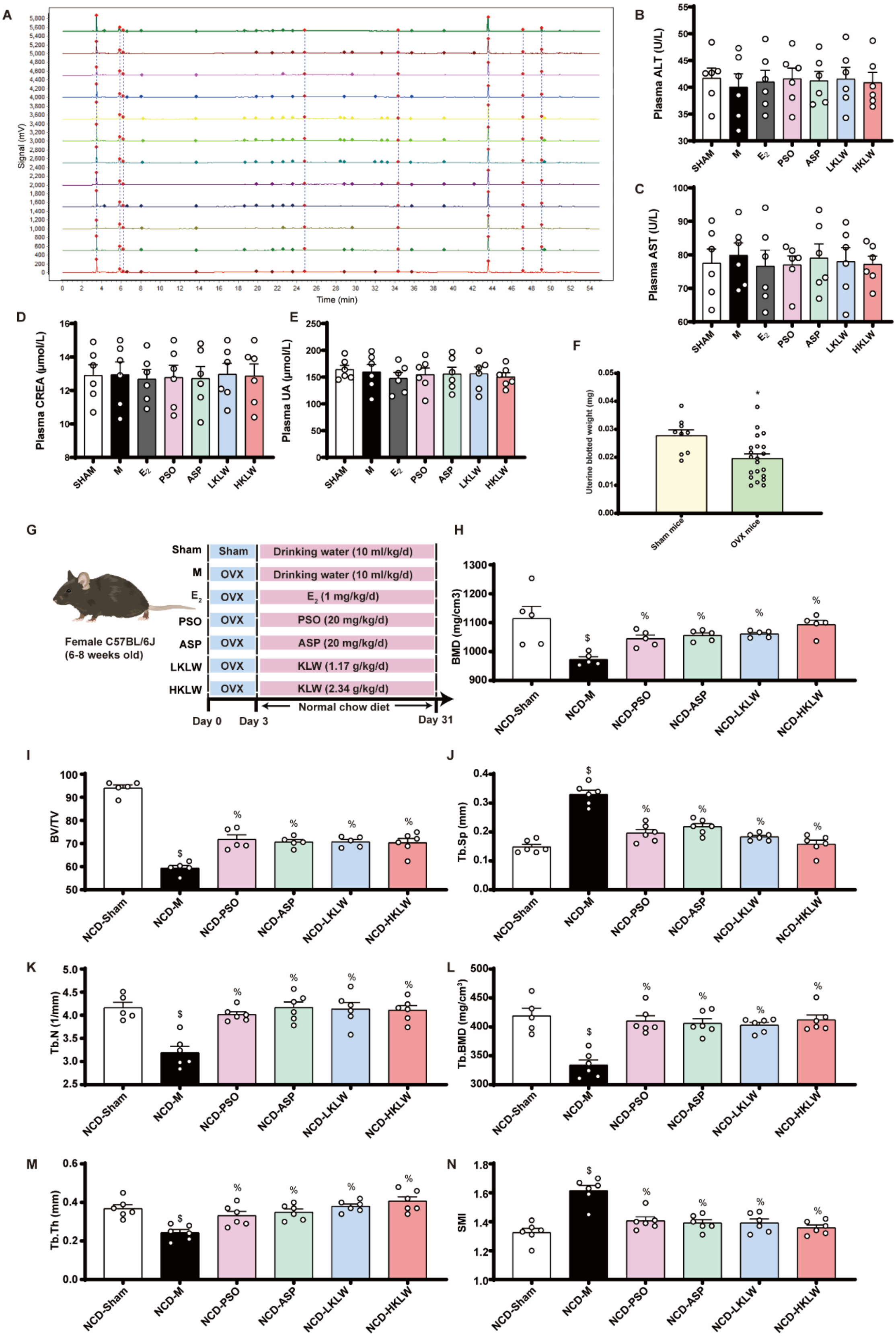
A
Signal (mV)
Time (min)
B
Plasma ALT (U/L)
SHAM
M
E_2
PSO
ASP
LKLW
HKLW
C
Plasma AST (U/L)
D
Plasma CREA (μmol/L)
E
Plasma UA (μmol/L)
F
Uterine blotted weight (mg)
Sham mice
OVX mice
G
Female C57BL/6J
(6-8 weeks old)
Sham
OVX
Drinking water (10 ml/kg/d)
E_2 (1 mg/kg/d)
PSO (20 mg/kg/d)
ASP (20 mg/kg/d)
KLW (1.17 g/kg/d)
KLW (2.34 g/kg/d)
Day 0
Day 3
Normal chow diet
Day 31
H
BMD (mg/cm3)
NCD-Sham
NCD-M
NCD-PSO
NCD-ASP
NCD-LKLW
NCD-HKLW
I
BV/TV
J
Tb.Sp (mm)
K
Tb.N (1/mm)
L
Tb.BMD (mg/cm³)
M
Tb.Th (mm)
N
SMI

Figure S1. KLW alleviated osteoporosis in OVX mice fed a normal chow diet.

(A)Common peak matching profile of 11 batches of KLW. (B) Alanine aminotransferase (ALT), (C) Aspartate aminotransferase (AST), (D) creatinine (CREA), and (E) uric acid (UA) were measured in each group. (F) Uterine blotted weight. Mice were randomly assigned to undergo either sham surgery or ovariectomy (OVX). After a 3-day postoperative recovery period, all animals were initiated on a normal-chow diet (NCD) which was maintained throughout the experimental period. Following diet initiation, mice received daily gavage treatments for 28 consecutive days according to their respective group assignments. The Sham, M, E_2, PSO, ASP, LKLW, and HKLW groups represent the sham-operated group, model group, estrogen group, psoralen group, asperosaponin VI group, low-dose KLW group, and high-dose KLW group, respectively. Uterine blotted weight. (G) Schematic diagram of animal grouping and drug administration regimen. (H-N) Quantitative analysis of bone microarchitectural parameters from micro-CT: (H) bone mineral density (BMD), (I) bone volume fraction (BV/TV), (J) trabecular separation (Tb.Sp), (K) trabecular number (Tb.N), (L) trabecular bone density (Tb.BMD), (M) trabecular thickness (Tb.Th), and (N) structure model index (SMI). All data are presented as the means ± SEM. Statistical analysis was performed using one-way ANOVA followed by Tukey's post hoc test: \$$P < 0.05$ compared to the NCD-Sham group, %$P < 0.05$ compared to the NCD-M group.

Figure. S2.

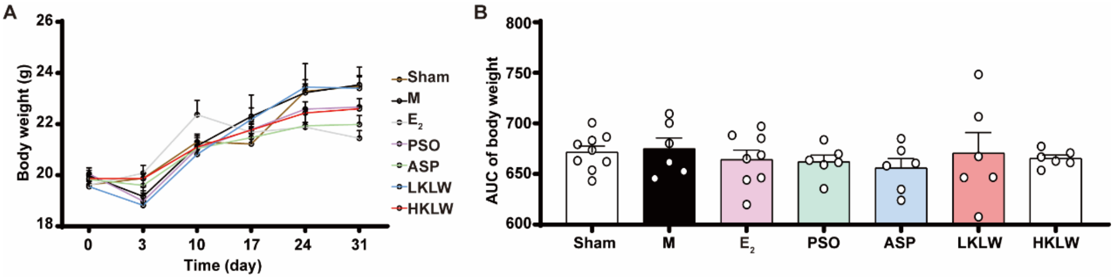

Figure S2. KLW does not significantly affect body weight in HFD-fed mice

Body weight was measured at each time point, and the area under the curve (AUC) was calculated for statistical analysis. (A) Body weight curve. (B) Area under the curve. All data are presented as the means ± SEM. Statistical analysis was performed using one-way ANOVA followed by Tukey's post hoc test.

Figure. S3.

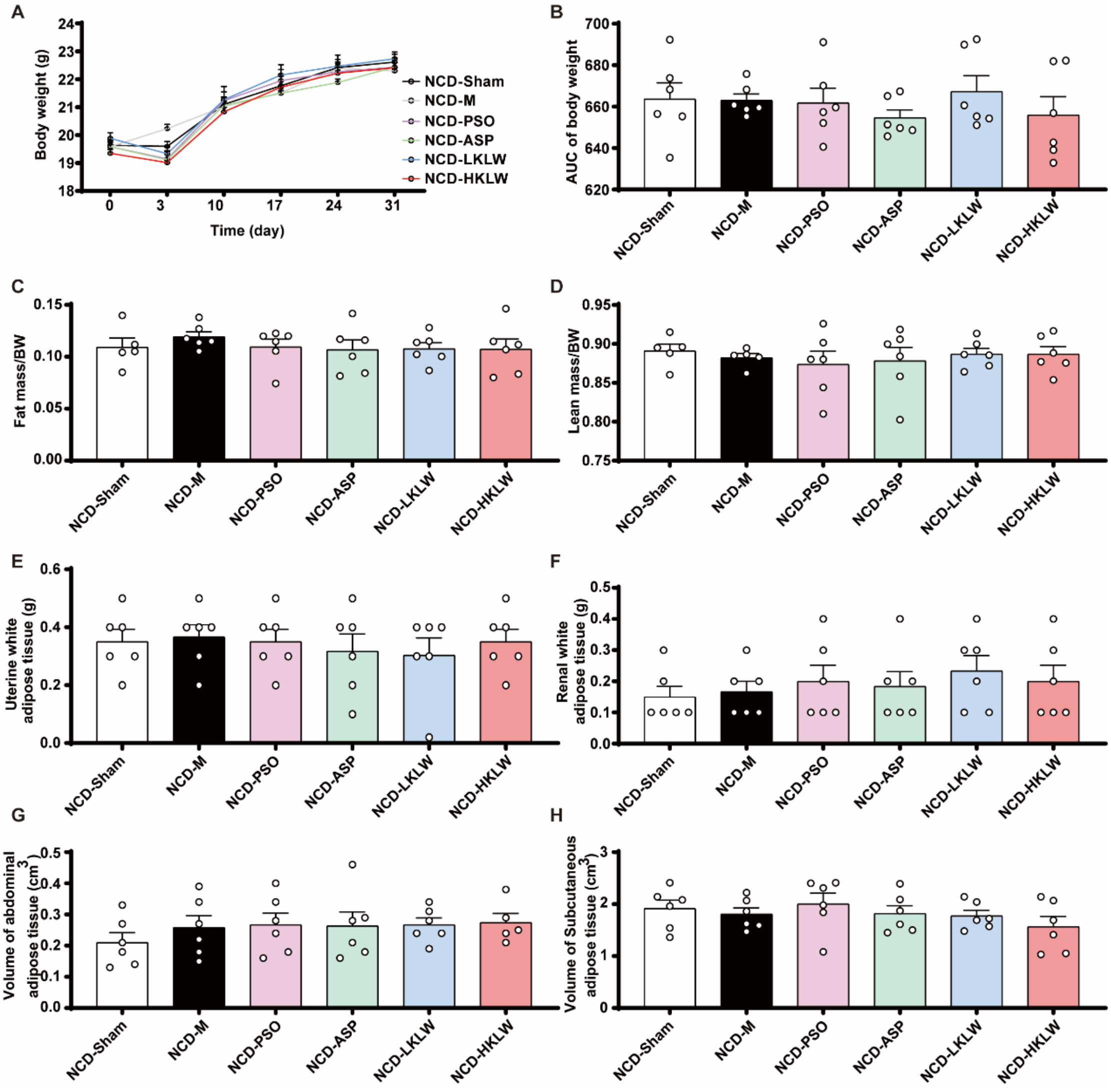

Figure S3. KLW does not alter body weight or body composition in ovariectomized mice under NCD.

Body weight was measured at each time point, and the area under the curve (AUC) was calculated for statistical analysis. (A) Body weight curve. (B) Area under the curve. (C) Body fat percentage. (D) Lean mass percentage. (E) uterine white adipose tissue weight. (F) Renal white adipose tissue weight. (G) Volume of abdominal adipose tissue. (H)

Volume of subcutaneous adipose tissue. All data are presented as the means ± SEM. Statistical analysis was performed using one-way ANOVA followed by Tukey's post hoc test.

Figure. S4

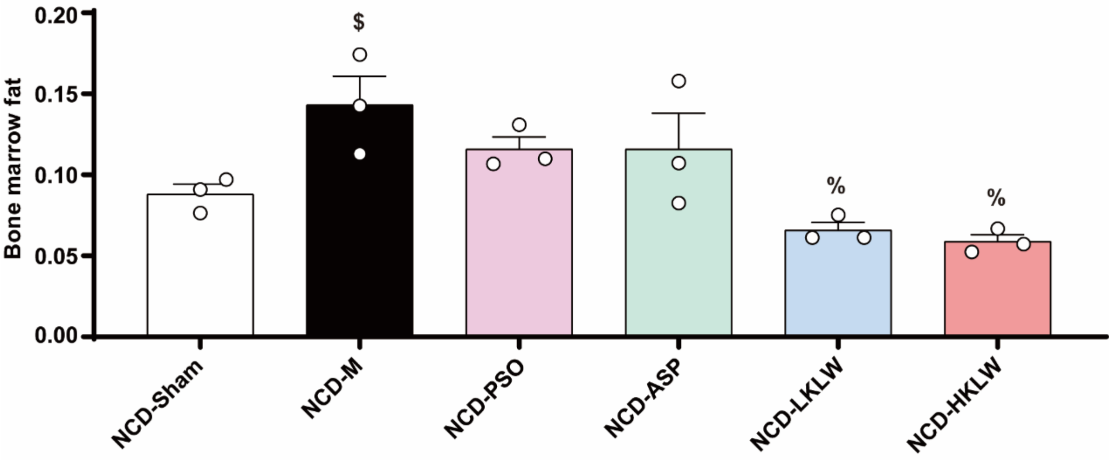

Figure S4. KLW treatment resulted in a reduction in bone marrow fat content in OVX mice under NCD.

Statistical results of the proton density fat fraction (PDFF) in the femoral bone marrow of mice. The values represent the average of bilateral measurements. All data are presented as the means ± SEM. Statistical analysis was performed using one-way ANOVA followed by Tukey's post hoc test: \$$P$ < 0.05 compared to the NCD-Sham group, %P < 0.05 compared to the NCD-M group.

Figure. S5

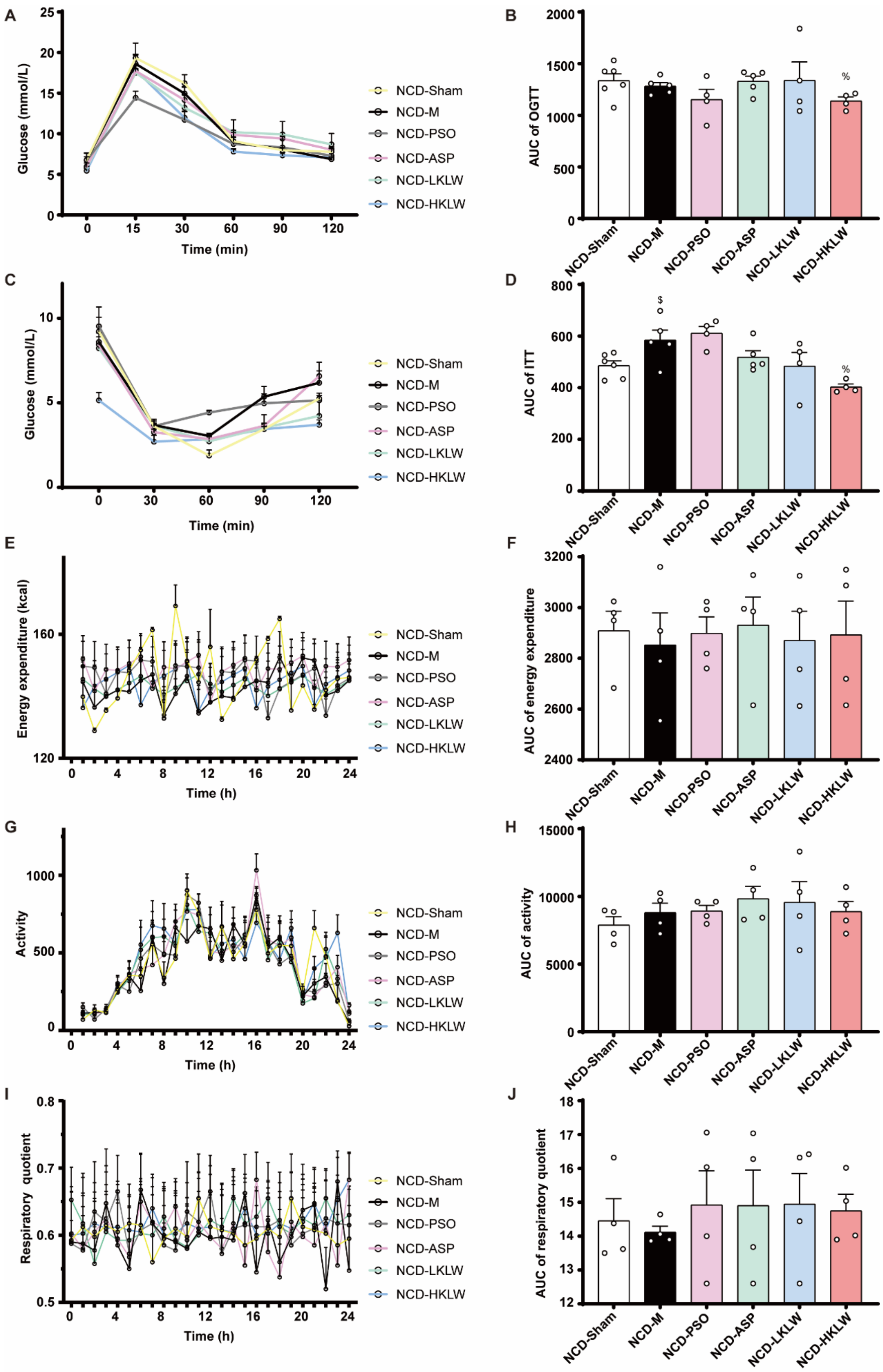
A
Glucose (mmol/L)
Time (min)
B
AUC of OGTT
C
Glucose (mmol/L)
Time (min)
D
AUC of ITT
E
Energy expenditure (kcal)
Time (h)
F
AUC of energy expenditure
G
Activity
Time (h)
H
AUC of activity
I
Respiratory quotient
Time (h)
J
AUC of respiratory quotient
NCD-Sham
NCD-M
NCD-PSO
NCD-ASP
NCD-LKLW
NCD-HKLW

Figure S5. Effects of KLW on metabolic homeostasis in NCD mice

(A-B) Oral Glucose Tolerance Test (OGTT) and AUC; (C-D) Insulin Tolerance Test, (ITT) and AUC; (E-F) Energy expenditure curve and AUC; (G-H) Activity curve and AUC; (I-J) Respiratory Exchange Ratio (RER) and AUC. All data are presented as the means ± SEM. Statistical analysis was performed using one-way ANOVA followed by Tukey's post hoc test: \$$P < 0.05$ compared to the NCD-Sham group, %$P < 0.05$ compared to the NCD-M group.

Table S1. Composition of KLW

Species	Chinese name	Part used	Family	Weight percent
Cyperus rotundus L	Xiangfu	Rhizome	Cyperaceae	15.7
Radix glycyrrhizae	Gancao	Rhizome	Papilionaceae	1.5
Radix cynanchi atrati	Baiwei	Root	Asclepiadaceae	3.0
Herba leonuri	Yimucao	Acrial part	Lamiaceae	3.0
Radix scutellariae	Huangqi	Root	Leguminosae	3.0
Celosia cristata	Jiguanhua	Inflorescence	Amaranthaceae	3.0
Radix ophiopogonis	Maidong	Earthnut	Liliaceae	3.0
Fructus Schisandrae chinensis	Wuweizi	Fruit	Magnoliaceae	3.0
Radix rehmanniae	Dihuang	Earthnut	Scrophulariaceae	3.0
Carthamus tinctorius L	Honghua	Flower	Asteraceae	3.0
Akebia quinata	Mutong	Caculis	Lardizabalaceae	2.1
Atractylodes macrocephala	Baizhu	Rhizome	Asteraceae	3.0
Halloysitum rubrum	Chishizhi	Kaolinite	Silicate	3.0
Poria cocos	Fuling	Sclerotium	Polyporaceae	3.0
Magnolia officinalis	Houpu	Dried bark, root bark, twig bark	Magnoliaceae	2.1
Cistanche salsa	Roucongrong	Fleshy stem	Orobanchaceae	3.0
Cynanchum otophyllum	Baishao	Root	Paeoniaceae	3.0
Nepeta cataria L	Jingjie	Stem leaf, spica	Labiatae	2.1
Cortex moutan	Mudanpi	Root bark	Ranunculaceae	3.0
Colla Corii Asini	Ejiao	Donkey skin	Equidae	3.0
Angelica sinensis	Danggui	Root	Lamiaceae	3.0
Ligusticum sinense Oliv	Gaoben	Rhizome, root	Umbelliferae	2.1
Panax ginseng C.A.Mey	Hongshen	Root	Araliaceae	3.0
Deerhorn Glue	Lujiaojiao	Antler	Cervidae	3.0

Fritillaria cirrhosa D. Don	Chuanbeimu	Stem	Liliaceae	3.0
Nacre Concha Margaritifera Usta	Moyao	Resin	Burseraceae	3.0
Amomum villosum Lour	Sharen	Fruit	Zingiberaceae	3.0
Corydalis	Yanhusuo	Tuber	Papaveraceae	3.0
Foeniculum vulgare	Xiaohuixiang	Fruit	Umbelliferae	3.0
Colla Carapacis et Plastri Testudinis	Guijiajiao	Carapace	Testudinidae	3.0
Ligusticum wallichii	Chuanxiong	Rhizome	Apiaceae Lindl	3.0

Table S2. Primers

**Gene**	**Forward (5’-3’)**	**Forward (5’-3’)**
Bmp2	AGCTGCAAGAGACACCCTTT	CATGCCTTAGGGATTTTGGA
Ocn	AGTCTGACAAAGCCTTCA	AAGCAGGGTTAAGCTCACA
Resistin	TCCTTGTCCCTGAACTGC	ACGAATGTCCCACGAGC
Fabp4	CCGATCCACTCCTTACCTCA	GCCACCGTGACCTTGTACTT
Pparγ	GCCCTTTACCACAGTTGATTTCT	GTGATTTGTCCGTTGTCTTTCCT
C/ebpβ	ACGGTGGACAAGCTGAGCG	CCTTGTGCTGCGTCTCCAGG
Runx2	CTCCGCCTGAGCCATGAAG	CACCAGTGATGATGCCATTCT
β-actin	GGCTGTATTCCCCTCCATCG	CCAGTTGGTAACAATGCCATGT
