# Supplementary figures and images for: Kun-Ling Wan Formula Ameliorates Postmenopausal Osteoporosis and Adipose Accumulation by Suppressing mTOR Signaling in Mesenchymal Stem Cells
Source: Pharmaceuticals (Basel). 2026 Apr 30;19(5):719. doi: 10.3390/ph19050719 (PMC13210056; doi:10.3390/ph19050719)

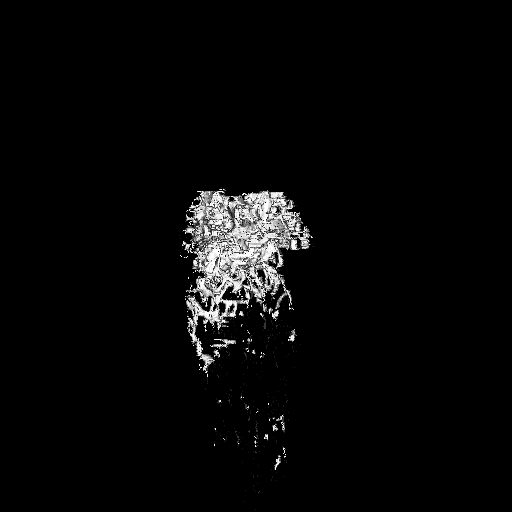

Supplement: Supplementary file 1 [file pharmaceuticals-19-00719-s001.zip › Video S1.GIF]

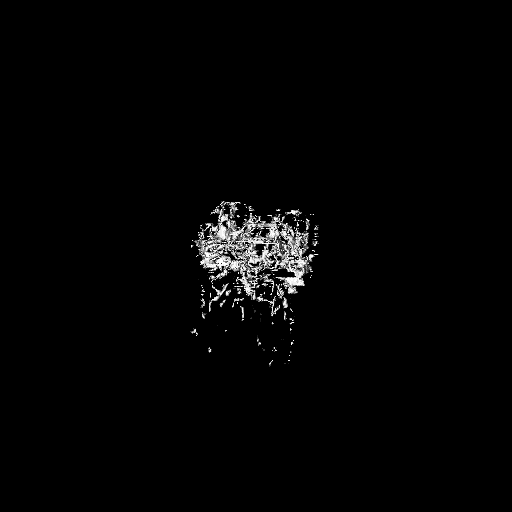

Supplement: Supplementary file 1 [file pharmaceuticals-19-00719-s001.zip › Video S2.gif]

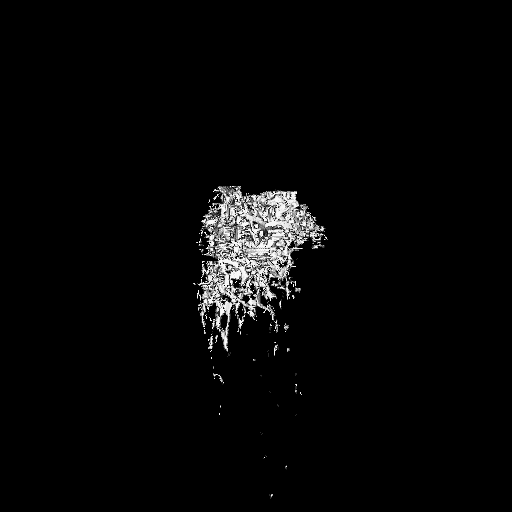

Supplement: Supplementary file 1 [file pharmaceuticals-19-00719-s001.zip › Video S3.GIF]

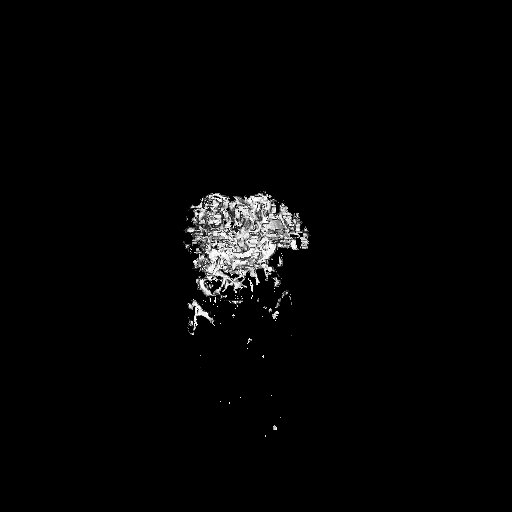

Supplement: Supplementary file 1 [file pharmaceuticals-19-00719-s001.zip › Video S4.GIF]

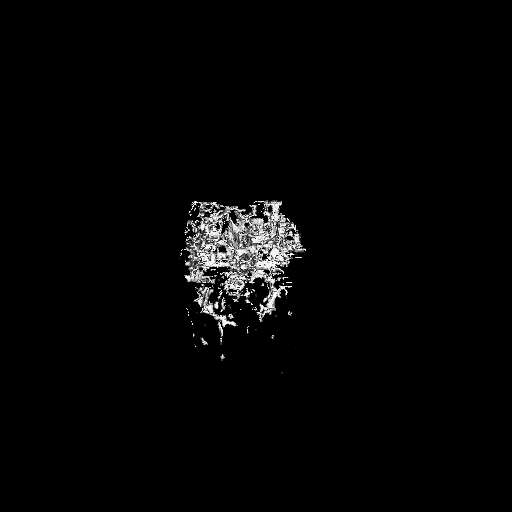

Supplement: Supplementary file 1 [file pharmaceuticals-19-00719-s001.zip › Video S5.GIF]

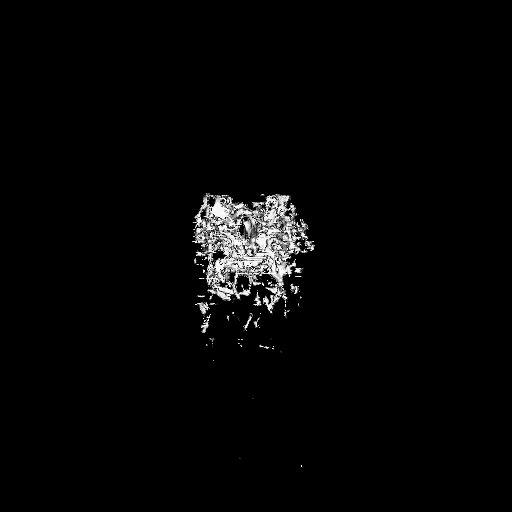

Supplement: Supplementary file 1 [file pharmaceuticals-19-00719-s001.zip › Video S6.gif]

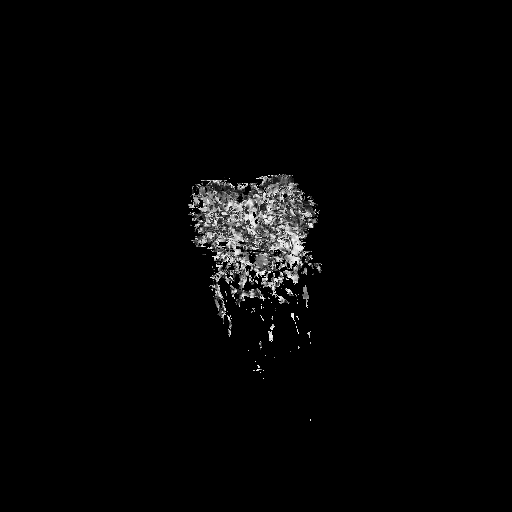

Supplement: Supplementary file 1 [file pharmaceuticals-19-00719-s001.zip › Video S7.GIF]
